# A CMTM6 Nanobody Overcomes EGFR‐TKI Resistance in Non‐Small Cell Lung Cancer

**DOI:** 10.1002/advs.202410945

**Published:** 2025-06-16

**Authors:** Lu Xia, Jichuan Wang, Hui Xue, Haimeng Li, Qinghua Li, Sen Qin, Chunyu Yu, Yanhua Liu, Yu Gao, Lingyun Li, Sudun Guan, Enrun Zheng, Feiya Suo, Lin He, Yongsheng Wang, Wenling Han, Yongfeng Shang, Yong Geng, Luyang Sun

**Affiliations:** ^1^ Department of Biochemistry and Molecular Biology School of Basic Medical Sciences Peking University International Cancer Institute State Key Laboratory of Natural and Biomimetic Drugs Peking University Health Science Center Beijing 100191 China; ^2^ Musculoskeletal Tumor Center Beijing Key Laboratory for Musculoskeletal Tumors Peking University People's Hospital Beijing 100044 China; ^3^ Department of Immunology School of Basic Medical Sciences Peking University Health Science Center NHC Key Laboratory of Medical Immunology Peking University Center for Human Disease Genomics Beijing 100191 China; ^4^ Department of Biochemistry and Molecular Biology School of Basic Medical Sciences Hangzhou Normal University Hangzhou 311121 China; ^5^ Department of Integration of Chinese and Western Medicine School of Basic Medical Sciences State Key Laboratory of Molecular Oncology Peking University Health Science Center Beijing 100191 China; ^6^ Thoracic Oncology Ward West China Hospital Cancer Center Sichuan University Chengdu 610041 China; ^7^ The CAS Key Laboratory of Receptor Research Shanghai Institute of Materia Medica Chinese Academy of Sciences Shanghai 201203 China

**Keywords:** CMTM6, EGFR, EGFR‐TKIs resistance, NSCLC, nanobody

## Abstract

Aberrant EGFR signaling drives non‐small cell lung cancer (NSCLC) development, and despite the success of tyrosine kinase inhibitor (TKI) therapies in treating NSCLC, TKI resistance remains a major obstacle. Here, we report that the chemokine‐like transmembrane protein CMTM6 is physically associated with EGFR. CMTM6 is shown to be co‐localized with EGFR in recycling endosomes that are marked by RAB11, thereby preventing EGFR from lysosome‐mediated degradation in NSCLC cells. The level of CMTM6 is elevated in NSCLC, and high expression of CMTM6 is associated with enhanced colocalization of CMTM6 with EGFR and RAB11 in NSCLC tumors and correlated with a poor prognosis in NSCLC patients. A CMTM6‐targeting nanobody is developed and administration of this agent leads to blocking of the CMTM6‐EGFR interaction, reduction of the EGFR protein level, and inhibition of the proliferation of TKI‐resistant NSCLC cells in vitro and suppression of the growth of EGFR‐TKI‐resistant NSCLC in both cell line‐derived xenografts and patient‐derived xenograft models. The study indicates that CMTM6 is a stabilizer of EGFR in endocytic trafficking and provides evidence to support targeting CMTM6 as a potential therapeutic strategy to overcome TKI resistance in NSCLC treatment.

## Graphical abstract

Overcoming EGFR‐TKI resistance remains a critical challenge in NSCLC treatment. This study identifies CMTM6 as a key regulator of EGFR stability and demonstrates that a novel anti‐CMTM6 nanobody disrupts the CMTM6‐EGFR interaction. Targeting CMTM6 restores EGFR degradation, suppresses tumor growth, and confers therapeutic benefit in both CDX and PDX models, offering a promising strategy against TKI‐resistant NSCLC.

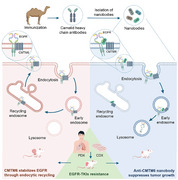

## 1 Introduction

Non‐small cell lung cancer (NSCLC) accounts for 85% to 90% of lung cancer cases,^[^ ^1^ ^]^ with lung adenocarcinoma (LUAD) as the most prevalent subtype (40%).^[^ ^2^ ^]^ Dysregulated epidermal growth factor receptor (EGFR) signaling is a prominent feature in multiple stages of NSCLC, especially in LUAD.^[^ ^3^ ^]^ Uncontrolled EGFR signaling has been ascribed to overexpression of the receptor or of its ligand(s), activating mutations in the EGFR kinase domain, dysregulation of signaling components downstream of the receptor, and escape from receptor degradation.^[^ ^4^ ^]^ Particularly, activating mutations such as L858R and exon 19 deletions account for 90% of mutations of EGFR in NSCLC.^[^ ^5^ ^]^

Over the years, EGFR‐targeted therapies with tyrosine kinase inhibitors (TKIs) such as erlotinib or gefitinib have been developed to greatly improve the outcome of patients with EGFR‐activating mutations in NSCLC.^[^ ^6^ ^]^ However, most patients eventually develop resistance to these 1st generation TKIs, often carrying EGFR/T790M mutation.^[^ ^7^ ^]^ Although osimertinib (AZD9291) was developed to address this resistance, new mutations like EGFR/C797S and EGFR/L718Q continue to emerge, highlighting the need for alternative therapeutic strategies to target EGFR signaling in EGFR‐TKI‐resistant NSCLC.^[^ ^8^ ^]^

EGFR signaling is tightly regulated by endocytic trafficking.^[^ ^4d^ ^]^ After ligand binding, EGFR undergoes endocytosis, followed by transport from the cell membrane to early endosomes where it is sorted and either recycled back to the cell membrane or targeted for lysosomal degradation.^[^ ^9^ ^]^ These processes involve Rab GTPases including RAB5, RAB11, and RAB7 that function directly in endocytic trafficking and fusion. In NSCLC, mutated EGFRs undergo ligand‐independent activation and aberrant endocytic trafficking. Specifically, their interaction with the E3 ubiquitin ligase CBL is impaired, leading to defective ubiquitination and degradation.^[^ ^10^ ^]^ The aberrant endocytic trafficking of EGFR is known to drive sustained and pathogenic signaling,^[^ ^4c^ ^]^ yet multiple questions regarding the pathway and regulation of the trafficking and endosomal signaling of EGFR in TKI‐resistant NSCLC remain unanswered.^[^ ^11^ ^]^

The chemokine‐like factor (CKLF)‐like MARVEL transmembrane domain‐containing member (CMTM) protein family includes CKLF and CMTM1‐8, featuring a structurally conserved MAL and related proteins for vesicle trafficking and membrane linking (MARVEL) domain.^[^ ^12^ ^]^ These proteins function in membrane dynamics and endocytosis, including endosome formation and scission progress through their interactions with other proteins or adaptors involved in endocytic trafficking.^[^ ^13^ ^]^ For example, CMTM6 has been reported widely linked to trastuzumab resistance in HER2‐positive breast cancer and in stabilizing cell surface CD58, enhancing bolster antitumor immune responses in melanoma.^[^ ^14^ ^]^ CMTM6 also interacts with PD‐L1 at the cell membrane and in recycling endosomes, thus preventing its lysosomal degradation, and hindering tumor‐specific T‐cell activity.^[^ ^15^ ^]^ Recent studies have shown that CMTM6 has been identified as a critical prognostic indicator in NSCLC, stabilizes PD‐L1 in the tumor microenvironment, with its coexpression alongside PD‐L1 correlating with improved outcomes in PD‐1 axis blockade therapy for NSCLC.^[^ ^16^ ^]^ However, whether CMTM6 regulates the endocytic trafficking of other proteins and the therapeutic potential of targeting CMTM6 in NSCLC warrant further investigation.

In the current study, we profiled the EGFR interactome and found that CMTM6 is physically associated with EGFR in NSCLC cells. We showed that the interaction of CMTM6 with EGFR leads to the stabilization of EGFR by preventing it from lysosome‐mediated degradation via recycling endosomes. Clinicopathological analysis indicated that the elevated level of CMTM6 is correlated with poor prognosis in NSCLC patients. We developed a CMTM6‐targeting nanobody and demonstrated that this agent was able to inhibit the proliferation of NSCLC cells in vitro and suppress the growth of NSCLC tumors in both TKI‐resistant NSCLC CDX and PDX models.

## 2 Results

### 2.1 CMTM6 is Physically Associated with EGFR in NSCLC Cells

**Figure 1:**
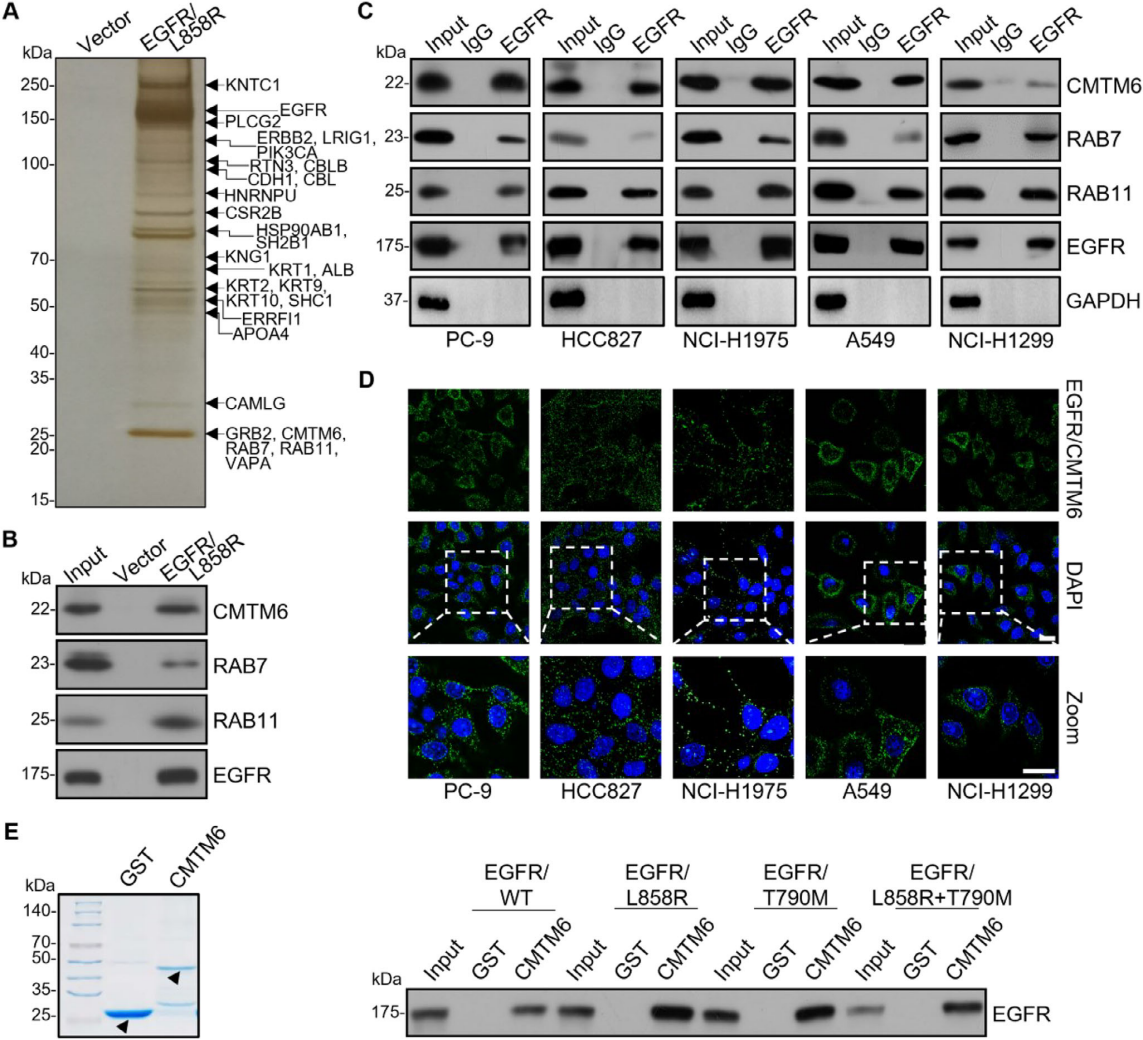
Physical interaction of CMTM6 with EGFR in NSCLC cells. A) Identification of EGFR‐interacting proteins by mass spectrometry. Cellular extracts from HEK293T cells stably expressing FLAG‐tagged EGFR/L858R were subjected to affinity purification with anti‐FLAG affinity columns. The bound proteins were eluted with FLAG peptides, resolved by SDS‐PAGE, and visualized by silver staining. Protein bands of interest were retrieved and analyzed by mass spectrometry. B) Immunoblotting analysis of the purified eluates. Purified eluates from Figure 1A were analyzed by immunoblotting using antibodies specific to the indicated proteins. C) Co‐immunoprecipitation in PC‐9, HCC827, NCI‐H1975, A549, and NCI‐H1299 cells with anti‐EGFR followed by immunoblotting with antibodies against the indicated proteins. D) Duolink proximity ligation assay (PLA) in PC‐9, HCC827, NCI‐H1975, A549, and NCI‐H1299 cells was performed using anti‐EGFR and anti‐CMTM6 antibodies. The PLA signals (green dots) indicate close proximity between the two proteins. Scale bar, 20 µm. E) GST pull‐down assays were performed with GST or with GST‐fused CMTM6 and in vitro transcribed/translated EGFR (wild‐type and variants bearing NSCLC patient mutations). Coomassie brilliant blue staining of the GST and GST‐fused CMTM6 are shown with arrows (left). The reaction was analyzed by immunoblotting with antibodies against EGFR (right).

Given the critical role of EGFR in the development and progression of NSCLC and considering that aberrant EGFR signaling is an eminent feature for EGFR‐TKI resistance in NSCLC,^[^ ^17^ ^]^ we wanted to search for potential targets that selectively modulate EGFR activity for possible more efficient treatments of NSCLC. To this end, we first profiled the EGFR interactome using epitope‐based proteomic screening combined with immunopurification and mass spectrometry. HEK293T cells, an established model for proteomic studies and for studying EGFR‐related processes,^[^ ^18^ ^]^ were stably transfected with FLAG‐tagged EGFR/L858R, the most frequent mutation in NSCLC.^[^ ^19^ ^]^ Cellular extracts were subjected to affinity purification using anti‐FLAG affinity columns, and the bound proteins were analyzed by mass spectrometry. Notably, EGFR/L858R was co‐purified with several previously reported EGFR‐interacting proteins such as GRB2, SHC1, CBL, and ERBB2^[^ ^20^ ^]^ (**Figure** **1**A). In addition, RAB7 and RAB11—small GTPase proteins integral to the constitutive endocytic trafficking system known to regulate EGFR intracellular transport^[^ ^4b^ ^]^—were also detected among the EGFR‐interacting proteins (Figure 1A). Interestingly, the transmembrane protein CMTM6, which was previously shown to function in the sorting and trafficking of membrane proteins within the endocytic trafficking network,^[^ ^14^, ^21^ ^]^ was also identified as an EGFR/L858R interacting protein (Figure 1A). Among the EGFR‐interacting proteins identified, ERBB2, though mutated or overexpressed in 2%–5% of lung cancers, is already an established target, making it less compelling for novel drug development.^[^ ^22^ ^]^ GRB2, SHC1, and CBL are cytoplasmic proteins without significant overexpression in lung cancer, limiting their therapeutic potential. In contrast, CMTM6, a transmembrane protein, is emerging as a key player in immune evasion in cancers like breast cancer and melanoma, promoting us to focus our research on CMTM6.^[^ ^14^ ^]^ The presence of CMTM6 in the EGFR interactome was confirmed by immunoblotting of the column‐bound proteins (Figure 1B).

Considering that the endocytic trafficking network has been implicated in the regulation of EGFR signaling and that CMTM6 has been explored as a therapeutic target against several malignancies including PD‐L1‐positive melanoma and HER2‐positive breast cancer,^[^ ^14^, ^15^, ^23^ ^]^ we next investigated whether and how CMTM6 influences the EGFR signaling in NSCLC. Pursuing this, we first validated the in vivo interaction of EGFR with CMTM6 using five representative NSCLC cell lines: PC‐9 and HCC827 with EGFR exon 19 deletion (EGFR exon 19del), NCI‐H1975 (EGFR/L858R+T790M), as well as the EGFR wild‐type cell line A549 and NCI‐H1299. Total proteins from these cells were extracted, and immunoprecipitation with a monoclonal antibody against EGFR followed by immunoblotting with antibodies against CMTM6, RAB7, and RAB11 showed that all of the examined proteins were efficiently co‐immunoprecipitated with EGFR from extracts of all examined NSCLC cell lines (Figure 1C). In addition, Duolink proximity ligation assays (PLA) were also performed in PC‐9, HCC827, NCI‐H1975, A549, and NCI‐H1299 cells to assess the proximity between CMTM6 and EGFR in situ. We detected discrete spots in the cell membrane and cytoplasm, supporting the interaction of CMTM6 with EGFR (Figure 1D). To further investigate the direct interaction between EGFR and CMTM6, glutathione S‐transferase (GST) pull‐down assays were performed with bacterially expressed GST or GST‐fused CMTM6 and in vitro transcribed/translated EGFR variants (wild‐type and the NSCLC clinically relevant L858R, T790M, and L858R/T790M mutants).^[^ ^24^ ^]^ Immunoblotting with the EGFR antibody showed that CMTM6 was capable of interacting with wild‐type as well as the mutated EGFR (Figure 1E). Collectively, these experiments indicate that CMTM6 is physically associated with both EGFR mutants and wild‐type EGFR in NSCLC cells.

### 2.2 CMTM6 Prevents EGFR from Being Targeted for Lysosome‐mediated Degradation via Recycling Endosomes in NSCLC Cells

It is well‐known that EGFR functions mainly at the cell membrane, where it binds to ligands, initiating dimerization, autophosphorylation, and downstream signaling.^[^ ^4d^ ^]^ To explore the pathophysiological significance of the physical association between CMTM6 and EGFR, we first investigated whether CMTM6 affects the level of EGFR associated with cell membranes. For this purpose, control and CMTM6‐knockdown PC‐9, HCC827, and NCI‐H1975 cells were stained with an anti‐EGFR fluorescent antibody for flow cytometry analysis. CMTM6 knockdown led to a significant decrease in the level of membrane EGFR (**Figure** **2**A), indicating that CMTM6 stabilizes membrane EGFR and suggesting that CMTM6 could influence EGFR signaling in NSCLC cells.

Figure 2Co‐localization of CMTM6 with EGFR in recycling endosomes with RAB11, preventing EGFR from lysosome‐mediated degradation. A) Flow cytometry of cell surface EGFR levels. HCC827, PC‐9, and NCI‐H1975 cells treated with control or CMTM6 siRNAs were collected for flow cytometry. B) Immunoblotting of lysates from PC‐9, NCI‐H1975, and HCC827 cells transfected with vector or FLAG‐CMTM6 or treated with control or CMTM6 siRNAs. Cellular extracts were prepared and analyzed by immunoblotting with antibodies against the indicated proteins. C) Immunoblotting analysis in PC‐9 cells treated with control or CMTM6 siRNAs (right). Cells were treated with DMSO for 8 h, MG132 for 8 h, or CQ for 4 h before being collected for immunoblotting. D) Double‐label immunofluorescence in PC‐9 cells fixed and stained with antibodies against CMTM6 (green) or EGFR (red), as well as markers (red) of recycling endosomes (RAB11), early endosomes (RAB5 and EEA1), late endosomes (RAB7), and lysosomes (LAMP1). Images were acquired using a confocal microscope. Scale bar, 10 µm. E) Multicolor immunofluorescence was performed to examine the colocalization of CMTM6 and EGFR in endosomes. PC‐9 cells were fixed and stained with antibodies against CMTM6 (red), EGFR (green), and a specific endosome marker (cyan) (RAB11, EEA1, RAB7, or LAMP1) simultaneously using tyramide signal amplification (TSA)‐based detection methods and multispectral imaging. Scale bar, 10 µm. F) Flow cytometry of cell surface EGFR levels under treatment with primaquine (PQ). PC‐9 cells treated with control or CMTM6 siRNAs were treated with DMSO or PQ for 4 h. Relative cell surface EGFR levels were analyzed by flow cytometry. Error bars represent the mean with SD, *p* values were calculated using a two‐tailed Student's t‐test (***p < 0.001, ns: not significant).

**Figure:**
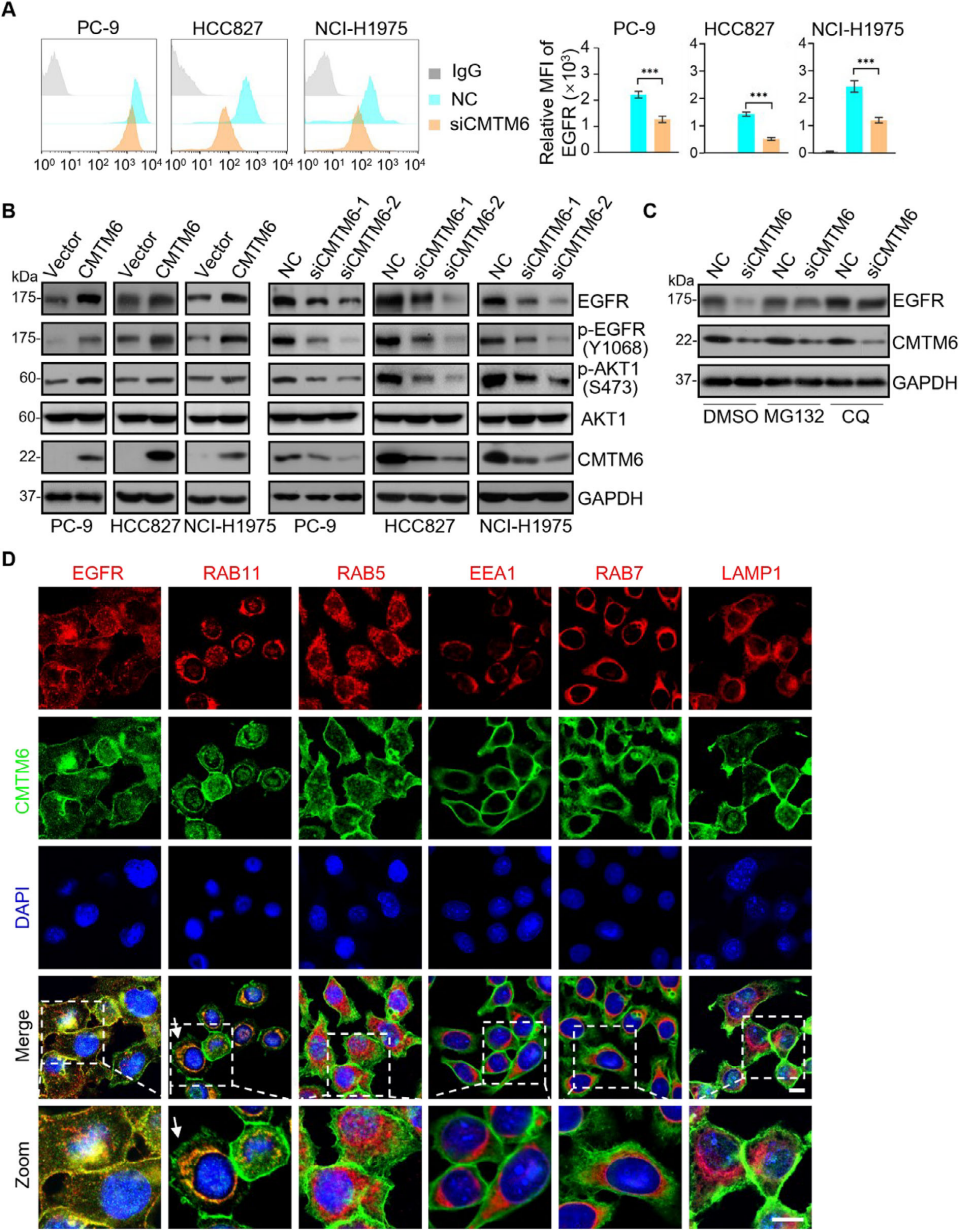

**Figure:**
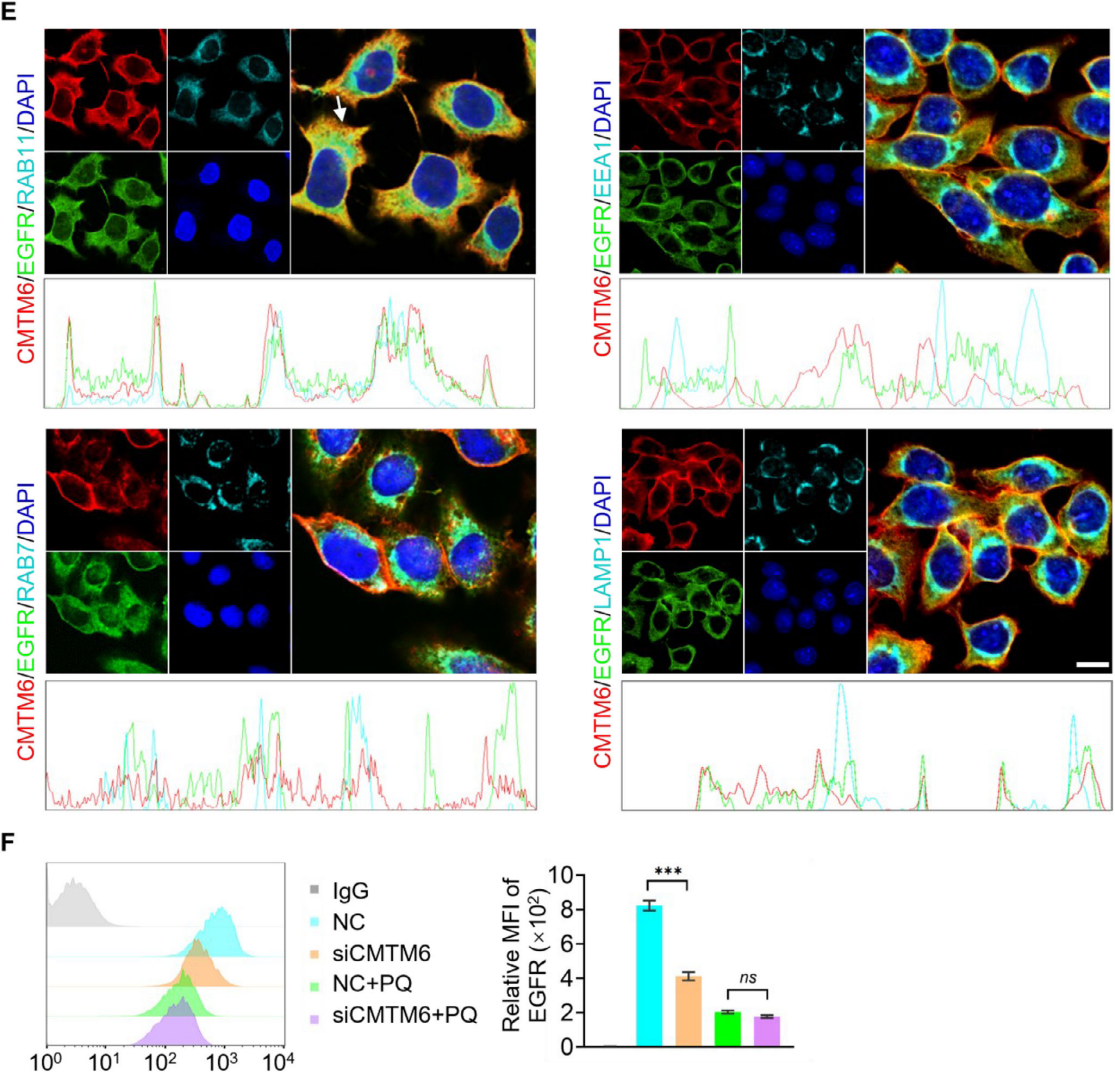

We next investigated the impact of CMTM6 on the steady‐state level of EGFR and its downstream signaling in NSCLC cells.^[^ ^25^ ^]^ PC‐9, HCC827, and NCI‐H1975 cells were either transfected with empty vector or CMTM6 expression plasmid or treated with control siRNAs or CMTM6 siRNAs. Immunoblotting with the EGFR antibody showed that CMTM6 overexpression was associated with an overt increase in EGFR expression and the phosphorylation levels of EGFR and AKT, whereas CMTM6 knockdown led to a marked decrease in EGFR expression and the phosphorylation levels of EGFR and AKT in all of these NSCLC cells (Figure 2B), indicating that CMTM6 promotes the stability of EGFR and activation of downstream signaling in NSCLC cells. To determine if the effect of CMTM6 on EGFR stability is mediated by proteasomal and/or lysosomal degradation, we exposed CMTM6‐knockdown PC‐9 cells to the proteasome inhibitor MG132 or the lysosomal inhibitor chloroquine (CQ). Immunoblotting showed that the inhibition of the lysosomal pathway, but not the proteasomal pathway, blocked the CMTM6 knockdown‐associated decrease in the EGFR level (Figure 2C), suggesting that CMTM6 acts to prevent lysosome‐mediated EGFR degradation.

As stated earlier, CMTM proteins are known to function in membrane dynamics and endocytic trafficking.^[^ ^13^, ^14^, ^21^ ^]^ To further understand the biological significance of the interaction between CMTM6 and EGFR, we next investigated whether CMTM6 modulates EGFR trafficking. EGFR trafficking involves a balance between recycling to the cell membrane via endocytic recycling and degradation via late endosomes during endocytic trafficking.^[^ ^9^ ^]^ We co‐stained CMTM6 and EGFR or CMTM6 and markers for endocytic compartments in PC‐9 cells, with RAB5 and EEA1 (Early Endosome Antigen 1) defining early endosomes, RAB11 defining recycling endosomes, and RAB7 defining late endosomes, or a marker for lysosomes

LAMP1 (Lysosome‐Associated Membrane Protein 1).^[^ ^26^ ^]^ Immunofluorescent microscopy showed that CMTM6 was co‐localized with EGFR both at the cell membrane and in the cytoplasm (Figure 2D). For the cytoplasmic CMTM6, the strongest signal was detected in recycling endosomes positively stained with RAB11, but not with early endosomes, late endosomes, or lysosomes (Figure 2D). Moreover, to enable simultaneous visualization of CMTM6, EGFR, and a specific endosomal marker, we employed tyramide signal amplification (TSA) technology for multicolor immunofluorescent staining in PC‐9 cells. Immunofluorescent microscopy detected a clear co‐localization of CMTM6 with EGFR and RAB11, whereas the co‐localization of CMTM6 and EGFR with EEA1, RAB7, or LAMP1 was not evident (Figure 2E), suggesting that CMTM6 is co‐localized with EGFR mainly in recycling endosomes, preventing EGFR from lysosome‐mediated degradation in NSCLC cells.

To further support this notion, we tested whether endocytic recycling is essential for the observed effect of CMTM6 on the EGFR level. Flow cytometry analysis of membrane‐localized EGFR in control and CMTM6‐knockdown PC‐9 cells showed that CMTM6 knockdown led to a significant decrease in the level of membrane‐localized EGFR, whereas treatment with primaquine (PQ), an inhibitor of endocytic recycling,^[^ ^27^ ^]^ effectively blocked the decrease in cell surface EGFR associated with CMTM6 knockdown (Figure 2F), implying that CMTM6 stabilizes EGFR at the cell membrane by facilitating its recycling through endocytosis. Collectively, these experiments demonstrate that CMTM6 promotes endocytic trafficking‐mediated stabilization of EGFR in NSCLC cells.

### 2.3 The CMTM6 Level is Elevated in NSCLC Tumors and is Inversely Correlated with NSCLC Patient Prognosis

**Figure 3:**
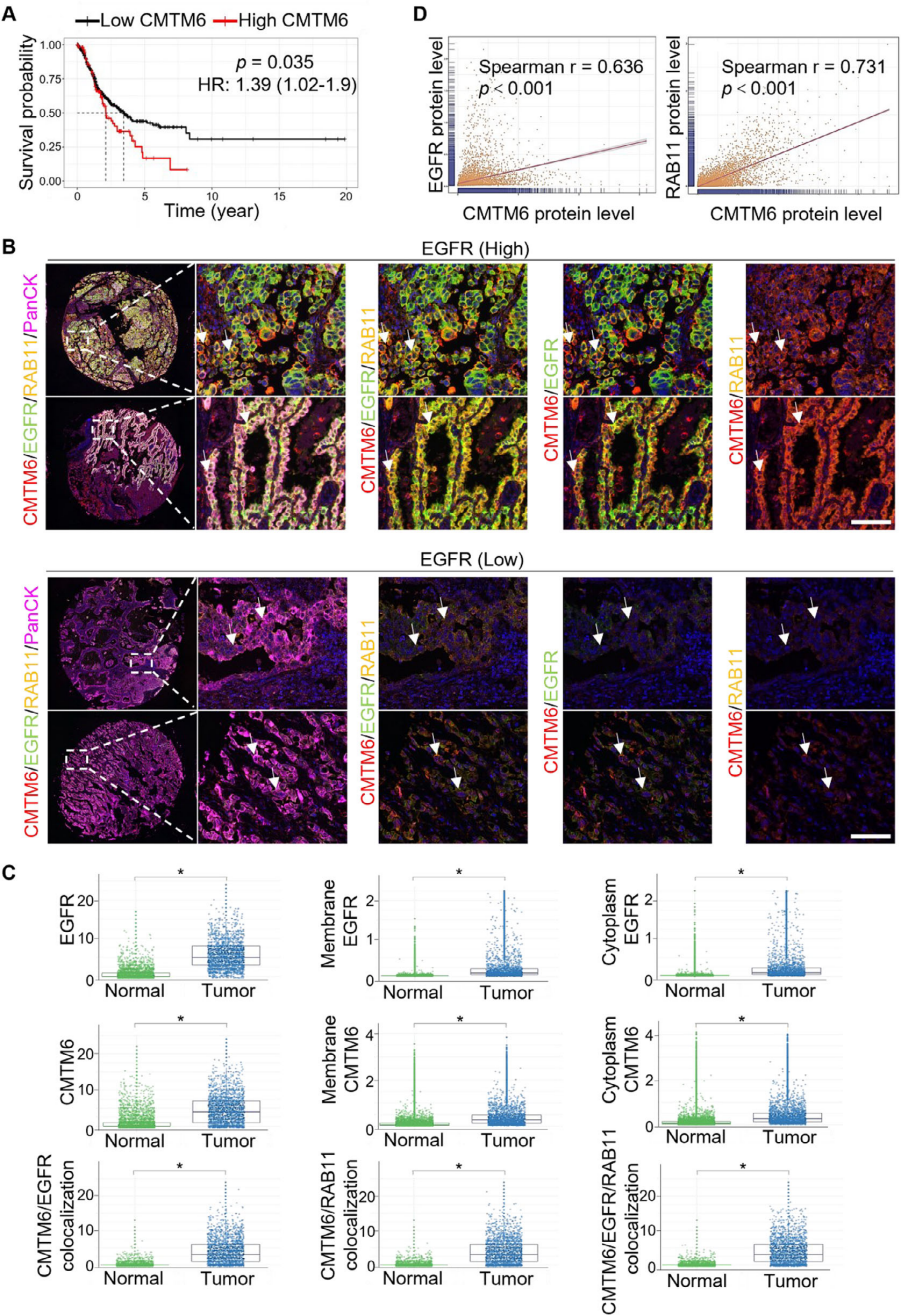
Elevated expression of CMTM6 correlates positively with EGFR and RAB11 levels in tumor regions of the NSCLC tissue array. A) Kaplan‐Meier survival analysis of the relationship between progression‐free interval and CMTM6 expression in TCGA‐NSCLC (*n* = 977) using an online survival analysis tool (http://www.biostatistics.online/topp/survival.php). B) Opal five‐color multiplexed immunohistochemistry (mIHC) was performed to compare the expression levels of CMTM6, RAB11, EGFR, and PanCK in 92 pairs of NSCLC and adjacent non‐tumor tissues. Tumor regions (PanCK+) and stromal regions (PanCK‐) were distinguished based on PanCK staining. Nuclei were stained with DAPI. Representative mIHC images are shown for patients with high (upper) and low (lower) EGFR expressions (*n* = 92). Scale bars, 50 µm. C) Quantitative analysis of EGFR (top) and CMTM6 (middle) levels were analyzed in NSCLC and adjacent non‐tumor tissues, including whole cell, membrane, and cytoplasmic levels. Co‐localization intensity of CMTM6 with RAB11, CMTM6 with EGFR, and CMTM6 with both EGFR and RAB11 (bottom) (*p < 0.05). D) Correlation analysis between the expression levels of CMTM6 and EGFR (left) or RAB11 (right) based on mIHC data. Survival curves were calculated with the Log‐rank (Mantel‐Cox) test. Pearson correlation was used. Error bars represent the mean with SD, and *p* values were calculated using a two‐tailed Student's t‐test (*p < 0.05).

To extend our observations to clinicopathologically relevant settings, we interrogated the NSCLC datasets in TCGA for CMTM6 expression employing an online tool (http://www.biostatistics.online/topp/survival.php) that separates samples into quartiles. The analysis indicated that the expression of CMTM6 is elevated, and high expression of CMTM6 is correlated with an unfavorable progression‐free interval (PFI) (*p* < 0.05) (**Figure** **3**A). We further performed multiplex immunohistochemistry (mIHC) on NSCLC tissue arrays to measure the CMTM6 level and to examine the association between EGFR and CMTM6 in tumors. The tissue arrays comprised NSCLC tumor samples paired with adjacent normal tissues from 92 NSCLC patients. Using multiplexed Opal fluorophores, we simultaneously evaluated five markers: CMTM6, EGFR, RAB11, DAPI (a nuclear dye), and Pan Cytokeratin (to discern tumor versus stromal cell identity) (Figure 3B).

CMTM6 expression was discernible in 43.23% of the tumor areas. Notably, the expression levels of CMTM6 and EGFR within whole cells, on the cell membrane, and in the cytoplasm, all exhibited a significant elevation in tumor areas compared to the adjacent stromal tissue (Figure 3C). Co‐expression profiling of CMTM6, EGFR, and/or RAB11 in the 92 cases indicated that the colocalization of CMTM6 with EGFR or RAB11 was detected in 29.75% and 29.88% of the tumor areas, respectively, and that the simultaneous colocalization of the three proteins was detected in 29.75% of the tumor areas (Figure 3C). Moreover, the intensity of the protein signals in these colocalizations exhibited a pronounced elevation in the tumor areas relative to the adjacent stromal tissue (Figure 3C). We further conducted a Spearman correlation analysis evaluating the level of CMTM6 in relationship to the level of EGFR or RAB11 and found a strong positive correlation of the CMTM6 expression with the expression of EGFR (*p* < 0.001, r = 0.636) and RAB11 (*p* < 0.001, r = 0.731) (Figure 3D). Collectively, these observations indicated that the expression of CMTM6 is elevated in NSCLC, and the level of its expression is inversely correlated with the prognosis of NSCLC patients, as well as with colocalization of EGFR in recycling endosomes in NSCLC tumor tissues, suggesting that CMTM6 is a potential therapeutic target for the treatment of NSCLC.

### 2.4 An Anti‐CMTM6 Nanobody Reduced the EGFR Level by Blocking the CMTM6‐EGFR Interaction in NSCLC Cells

**Figure 4:**
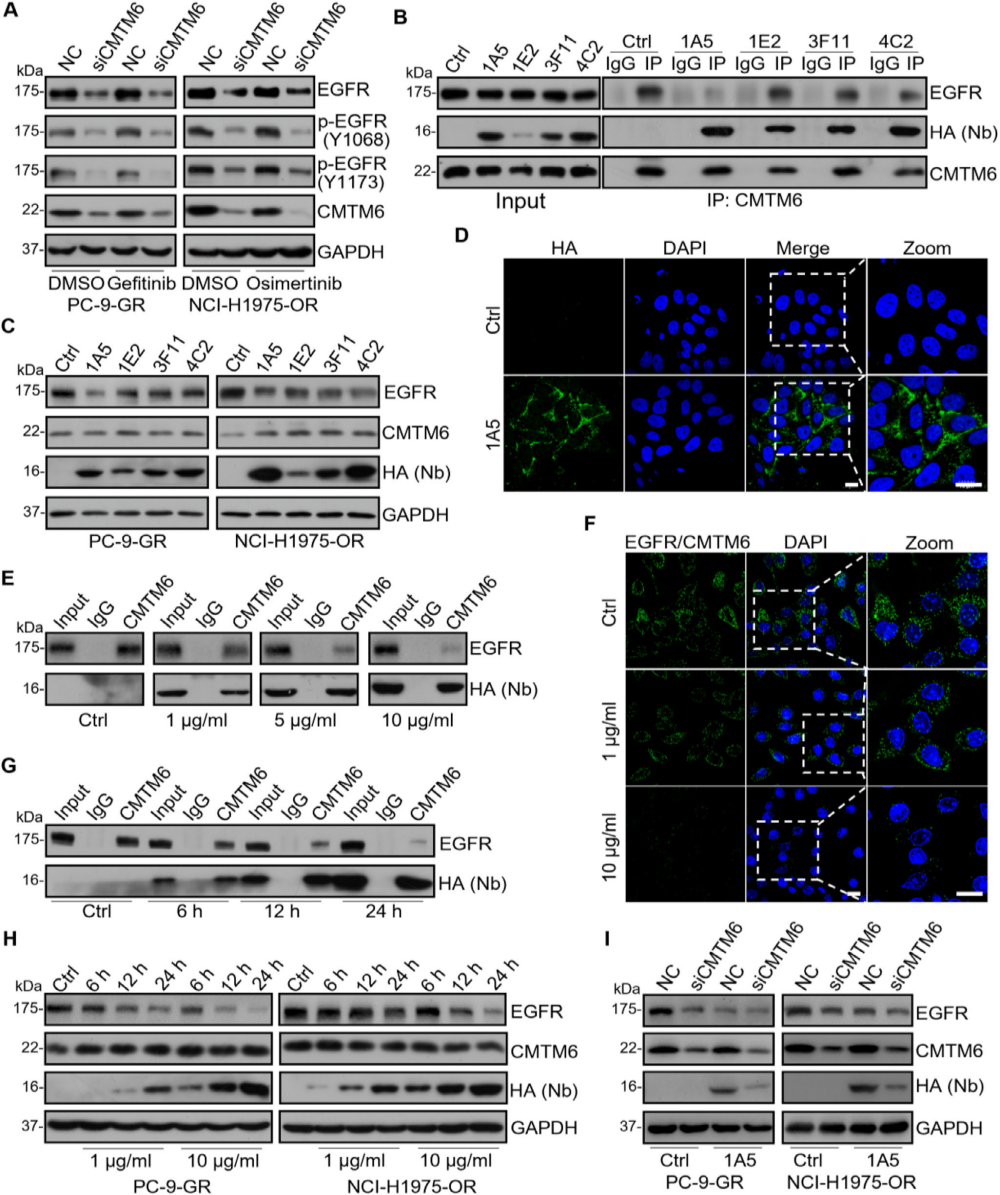
An anti‐CMTM6 nanobody decreases EGFR levels by blocking CMTM6 from binding to EGFR in NSCLC Cells. A) Immunoblotting analysis of lysates from PC‐9‐GR and NCI‐H1975‐OR cells treated with control or CMTM6 siRNAs under treatment of DMSO, gefitinib, or osimertinib for 24 h. Cellular extracts were prepared, and immunoblotting was performed with the antibodies against the indicated proteins. B) PC‐9‐GR cells were treated with IgG control (Ctrl) or anti‐CMTM6 HA‐tagged nanobodies (1A5, 1E2, 3F11, and 4C2) for 12 h at the concentration of 10 µg mL^−1^. Cells were treated with CQ for 4 h before being collected for immunoblotting. Co‐immunoprecipitation with anti‐CMTM6 was followed by immunoblotting with antibodies against the indicated proteins. Nb represents the nanobody. C) Immunoblotting of lysates from PC‐9‐GR and NCI‐H1975‐OR cells treated with control or HA‐tagged nanobodies (1A5, 1E2, 3F11, and 4C2). Cellular extracts were prepared, and immunoblotting was performed with the antibodies against the indicated proteins. D) Immunofluorescence to monitor the localization of HA‐tagged 1A5 nanobody. PC‐9 cells were treated with control or nanobody 1A5 for 12 h and were fixed and stained with antibodies against HA. Scale bar, 10 µm. E) Co‐immunoprecipitation in PC‐9‐GR cells treated with control or varying concentrations of HA‐tagged 1A5 nanobody (1, 5, or 10 µg mL^−1^) for 24 h. Immunoprecipitation was performed with anti‐CMTM6 followed by immunoblotting. F) Duolink proximity ligation assay in PC‐9‐GR cells treated with control or nanobody 1A5 for 12 h. The assay was performed with anti‐EGFR and anti‐CMTM6 antibodies. Scale bar, 20 µm. G) Co‐immunoprecipitation in PC‐9‐GR cells treated with control or 10 µg mL^−1^ HA‐tagged 1A5 nanobody for 6, 12, or 24 h with anti‐CMTM6 followed by immunoblotting. H) Immunoblotting of lysates from PC‐9‐GR cells treated with control or CMTM6 siRNAs were treated with 1 or 10 µg mL^−1^ 1A5 for 6, 12, or 24 h. Cellular extracts were prepared, and immunoblotting was performed with the antibodies against the indicated proteins. I) Immunoblotting analysis of lysates from PC‐9‐GR and NCI‐H1975‐OR cells treated with control or CMTM6 siRNAs under treatment of control or 10 µg mL^−1^ 1A5 for 24 h. Cellular extracts were prepared, and immunoblotting was performed with the antibodies against the indicated proteins. Data are representative of at least three independent experiments.

As aberrant EGFR signaling is critically involved in the development and progression of NSCLC, tyrosine kinase inhibitors (TKIs) have been developed to successfully treat NSCLC patients in the past decades. Unfortunately, however, acquired resistance to TKI therapies also occurs and poses a significant challenge in combating this disease.^[^ ^28^ ^]^ Given the activity of CMTM6 in stabilizing EGFR in NSCLC cells, we next investigated the effect of CMTM6 on the treatment of NSCLC with TKIs. To this end, we first generated stable EGFR TKI‐resistant NSCLC cell lines, gefitinib‐resistant PC‐9 (GR) (EGFR exon 19del+/T790M) and osimertinib‐resistant NCI‐H1975 (OR) (EGFR/L858R+T790M+C797S), by exposing PC‐9 and NCI‐H1975 cells to increasing concentrations of gefitinib or osimertinib, respectively, over 6 months.^[^ ^29^ ^]^ As Y1068 and Y1173 in the EGFR sequence are two tyrosine phosphorylation sites representing the functional activation of EGFR in EGFR‐mutated NSCLC,^[^ ^28^ ^]^ we performed immunoblotting using anti‐Y1068 and anti‐Y1173 in PC‐9‐GR and NCI‐H1975‐OR cells and found unchanged tyrosine phosphorylation levels (**Figure** **4**A), indicative of successful establishment of TKI resistance in these cell lines. In addition, immunoblotting with anti‐EGFR in PC‐9‐GR and NCI‐H1975‐OR cells treated with control siRNAs or CMTM6 siRNAs showed that CMTM6 knockdown was associated with a decrease in the EGFR protein level in TKI‐resistant cells (Figure 4A).

We next evaluated the potential of targeting CMTM6 based on a set of single‐domain antibodies containing camelid VHHs and HA‐tag, also referred to as nanobodies, that we developed.^[^ ^30^ ^]^ The binding efficacy of these nanobodies to human CMTM6 was systematically validated. We first examined the affinity of various anti‐CMTM6 nanobodies, including 1A5, 1E2, 3F11, and 4C2 in NSCLC cells. PC‐9‐GR cells were treated with nanobodies for 24 h and with CQ for 4 h prior to immunoprecipitation with an antibody against CMTM6 followed by immunoblotting with an antibody against HA‐tag: the nanobodies bound to CMTM6 efficiently, with 1A5 and 4C2 exhibiting the strongest affinity (assessed via HA tag levels) (Figure 4B). In addition, immunoblotting in PC‐9‐GR cells with an antibody against EGFR of the CMTM6‐bound proteins showed that nanobody 1A5 had the strongest effect in weakening the interaction between CMTM6 and EGFR (Figure 4B). Moreover, immunoblotting using the antibody against EGFR in PC‐9‐GR and NCI‐H1975‐OR cells treated with these nanobodies showed that the 1A5 treatment was associated with the most pronounced decrease in the EGFR protein level (Figure 4C). We thus focused on 1A5 for the following studies.

Immunofluorescent microscopy using an anti‐HA tag in PC‐9‐GR cells under 1A5 treatment showed that 1A5, upon binding to cells, was localized at the cell membrane and in the cytoplasm (Figure 4D). We further investigated the concentration‐ and time‐dependent effects of nanobody 1A5 on the interaction between CMTM6 and EGFR. For this, PC‐9‐GR cell lines were first treated with varying concentrations of 1A5, followed by immunoprecipitation with the antibody against EGFR in the presence of CQ. Immunoblotting with the antibody against CMTM6 showed that as the concentration of nanobody 1A5 increased, the CMTM6‐EGFR interaction was weakened, indicating a concentration‐dependent inhibition by 1A5 (Figure 4E), which competes with EGFR for CMTM6 binding. DuoLink assays in PC‐9‐GR cells treated with different concentrations of 1A5 confirmed the concentration‐dependent inhibition by 1A5 (Figure 4F). PC‐9‐GR cells were then treated with 1A5 for 6, 12, and 24 h, co‐immunoprecipitation experiments showed that as the duration of nanobody 1A5 treatment increased, the CMTM6‐EGFR interaction was also weakened (Figure 4G), suggesting a time‐dependent inhibition by 1A5. These observations support the notion that treatment of NSCLC cells with nanobody 1A5 blocks the interaction of CMTM6 and EGFR.

To substantiate this and to investigate the biological consequence of the blocked interaction of CMTM6 and EGFR by 1A5, we next measured the level of EGFR in NSCLC cells treated with 1A5. For this, control or CMTM6‐knockdown PC‐9‐GR and NCI‐H1975‐OR cells were treated with different concentrations of nanobody 1A5 at different times. Immunoblotting with anti‐EGFR showed that in control cells, as the nanobody 1A5 concentration and duration increased, the EGFR protein level decreased (Figure 4H), whereas in CMTM6‐knockdown cells, the decrease in the EGFR protein level upon 1A5 treatment was not evident (Figure 4I). These observations indicate that nanobody 1A5 exerts a concentration‐ and time‐dependent inhibition of the CMTM6‐EGFR interaction, resulting in a reduction of the EGFR protein level.

### 2.5 The Anti‐CMTM6 Nanobody 1A5 Inhibits the Proliferation of EGFR‐TKI‐Resistant NSCLC Cells

**Figure 5:**
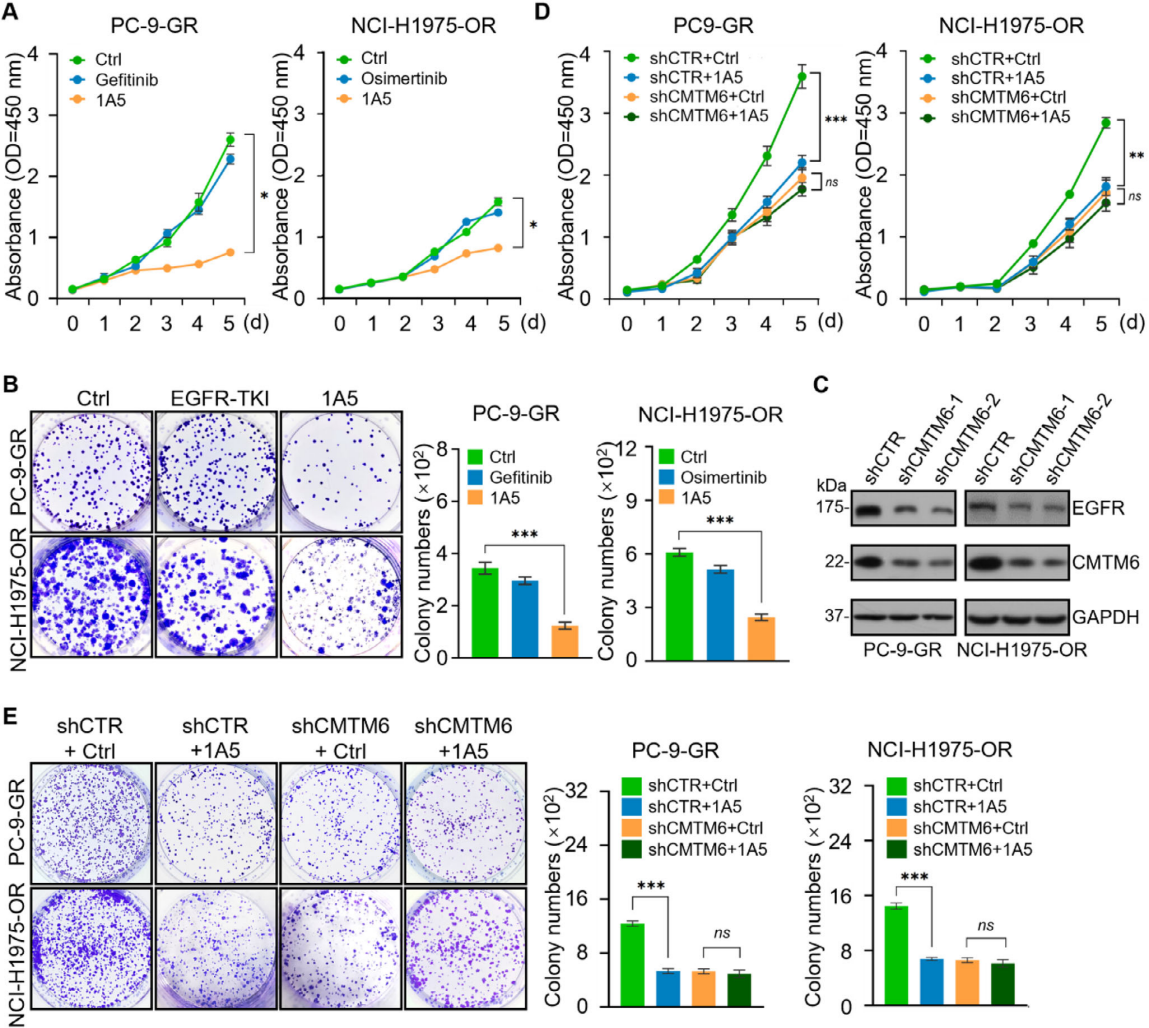
The anti‐CMTM6 nanobody overcomes resistance to EGFR‐TKI in NSCLC cells. A) Cell Counting Kit (CCK) assays were performed to measure the growth of PC‐9‐GR and NCI‐H1975‐OR cells cultured in the presence of control (DMSO and IgG), EGFR‐TKI (gefitinib or osimertinib), or nanobody 1A5. B) Colony formation assays were performed to measure the growth of PC‐9‐GR and NCI‐H1975‐OR cells cultured in the presence of control, EGFR‐TKI (gefitinib or osimertinib), or nanobody 1A5 for 14 days. Then cells were stained with crystal violet and colony numbers were counted. C) The efficiency of knockdown in PC‐9‐GR and NCI‐H1975‐OR cells transduced with lentiviruses carrying the indicated specific shRNAs was verified by immunoblotting. D) CCK assays were performed to measure the growth of PC‐9‐GR and NCI‐H1975‐OR cells infected with lentiviruses carrying CMTM6 shRNA and treated with either a control or nanobody 1A5. E) Colony formation assays were performed to measure the growth of PC‐9‐GR and NCI‐H1975‐OR cells infected with lentiviruses carrying CMTM6 shRNA, followed by treatment with either a control or nanobody 1A5. Error bars represent the mean with SD, *p* values were calculated using a two‐tailed unpaired Student's t‐test (*p < 0.05, **p < 0.01, ***p < 0.001, ns: not significant).

We next tested whether 1A5 could inhibit the proliferation of TKI‐resistant NSCLC cells. Cell proliferation measurements in PC‐9‐GR and NCI‐H1975‐OR cells using Cell Counting Kit (CCK) showed that 1A5 had a significant inhibitory effect on the proliferation of both these cell lines, whereas neither gefitinib nor osimertinib affected the proliferation of these cells (**Figure** **5**A). In addition, colony formation assays in PC‐9‐GR and NCI‐H1975‐OR cells showed that 1A5 treatment led to a significant decrease in the colony number, whereas such a decrease was not evident in cells treated with gefitinib or osimertinib (Figure 5B). These observations indicate that the anti‐CMTM6 nanobody 1A5 inhibits the proliferation of TKI‐resistant NSCLC cells.

To test whether the inhibitory effect of the nanobody 1A5 on the proliferation of TKI‐resistant NSCLC cells is mediated by blocking the CMTM6‐EGFR interaction, we conducted depletion of CMTM6 to assess potential interference with the inhibition of NSCLC cell proliferation by 1A5. CMTM6‐knockdown PC‐9‐GR or NCI‐H1975‐OR cell lines were constructed via lentivirally delivered short hairpin RNAs (shRNAs). Immunoblotting confirmed the effective depletion of CMTM6 with the expected outcome that CMTM6 knockdown led to decreased EGFR protein levels (Figure 5C). CMTM6‐knockdown PC‐9‐GR or NCI‐H1975‐OR cells were treated with control or nanobody 1A5 for CCK cell proliferation and colony formation assays. The CCK assays showed that 1A5 strongly inhibited the proliferation of control cells, while it had a limited effect on the proliferation of CMTM6‐knockdown cells (Figure 5D). Colony formation assays yielded similar results (Figure 5E). Together with the above‐described observations, these results support the notion that the anti‐CMTM6 nanobody 1A5 inhibits the proliferation of EGFR‐TKI‐resistant cells by blocking the proliferation‐promoting effect of CMTM6.

### 2.6 The Anti‐CMTM6 Nanobody Suppresses the Growth of EGFR‐TKI‐Resistant NSCLC Tumors

Given the capability of the nanobody 1A5 to inhibit the proliferation of EGFR‐TKI‐resistant NSCLC cells, we next examined the effect of 1A5 on the growth of NSCLC tumors. To this end, both TKI‐resistant NSCLC cell‐line‐derived xenograft (CDX) and patient‐derived tumor xenograft (PDX) mouse models were generated and utilized (**Figure** **6**A). For CDX models, TKI‐resistant PC‐9‐GR or NCI‐H1975‐OR cells were subcutaneously injected into 6‐week‐old female BALB/c nude mice (*n* = 6), with subcutaneous injection of 1A5 for seven times. We found that administration of 1A5 resulted in a significantly suppressed growth of the primary tumors (Figure 6B; and Figure S1A, Supporting Information), and immunostaining of sections from harvested tumors for Ki‐67 showed that 1A5 treatment was associated with a decreased cell proliferation (Figure 6C), whereas gefitinib or osimertinib (administered via gavage for two weeks) had limited effects on the growth of the tumors and the proliferation of the TKI‐resistant cells as expected (Figure 6B,C).

Figure 6Anti‐CMTM6 nanobody suppresses the growth of EGFR‐TKI‐Resistant NSCLC tumor in Vivo. A) Establishment of TKI‐resistant CDX (cell line‐derived xenograft) and PDX (patient‐derived xenograft) tumor models. For CDX models, PC‐9‐GR or NCI‐H1975‐OR cells were subcutaneously injected into 6‐week‐old female BALB/c nude mice (*n* = 6 per group), respectively. For PDX models, patient‐derived tumor tissues were subcutaneously implanted into 6‐week‐old female NCG mice (*n* = 6). Gefitinib or osimertinib was administered by gavage daily for two weeks, and hIgG1 or 1A5‐Fc was administered intraperitoneally for seven doses. B) Measurement of primary CDX tumor size at 6 weeks post‐injection. Images of primary tumors are shown. C) Immunohistochemical staining of CDX tumor sections for Ki‐67. Scale bars, 50 µm. The tumor proliferation index (percentage of Ki‐67^+^ cells) was counted. D) Measurement of primary PDX tumor size at 50 days post‐implantation. Representative images of primary tumors are shown. E) Immunoblotting analysis of TKI‐resistant PDX tumors. Protein and phosphorylation levels of EGFR were determined. F) Immunohistochemical staining of PDX tumor sections for Ki‐67. Scale bars, 50 µm. The proliferation index (percentage of Ki‐67^+^ cells) was quantified. Error bars represent the mean with SD, *p* values were calculated using a two‐tailed unpaired Student's t‐test (***p < 0.001).

**Figure:**
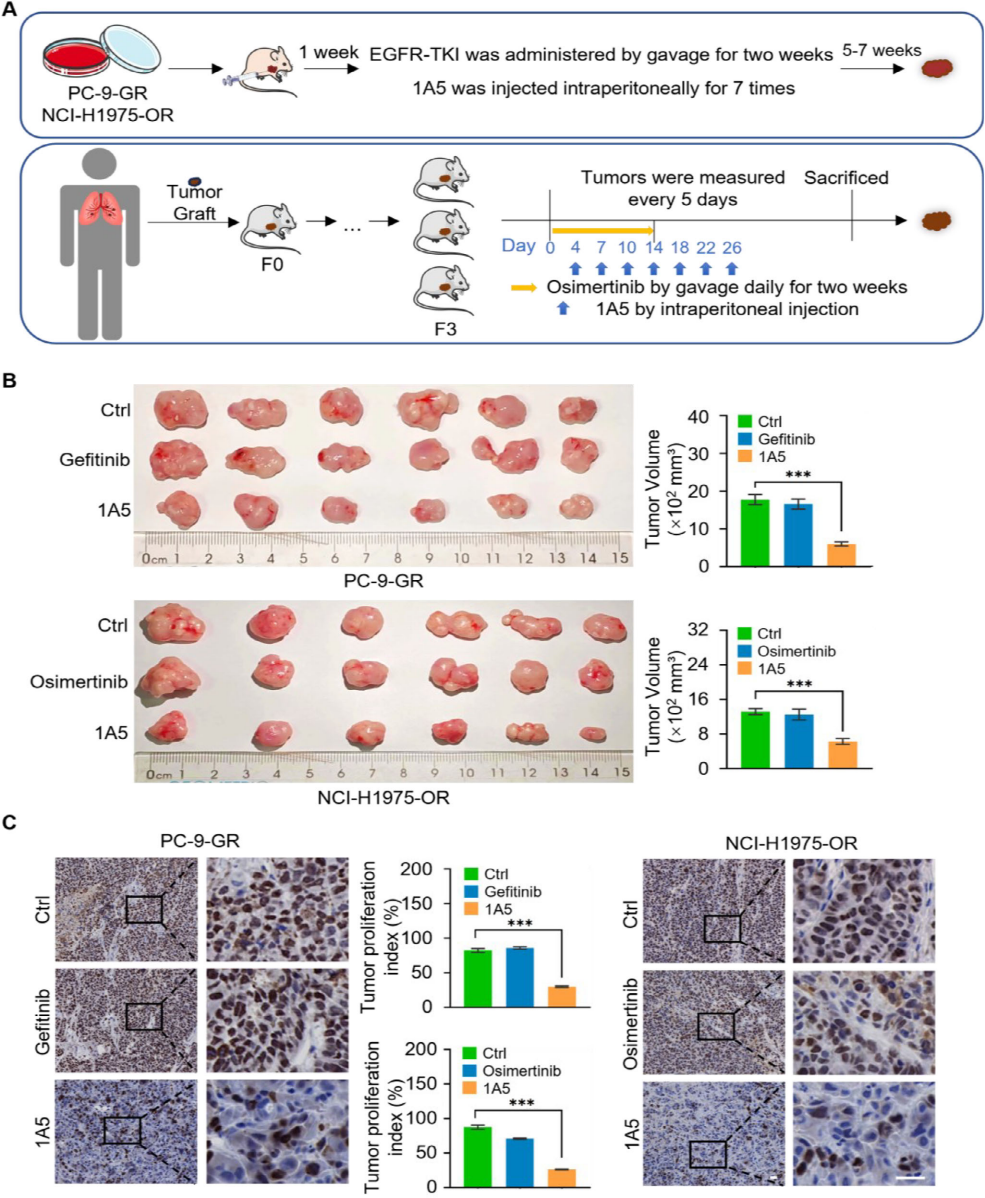

**Figure:**
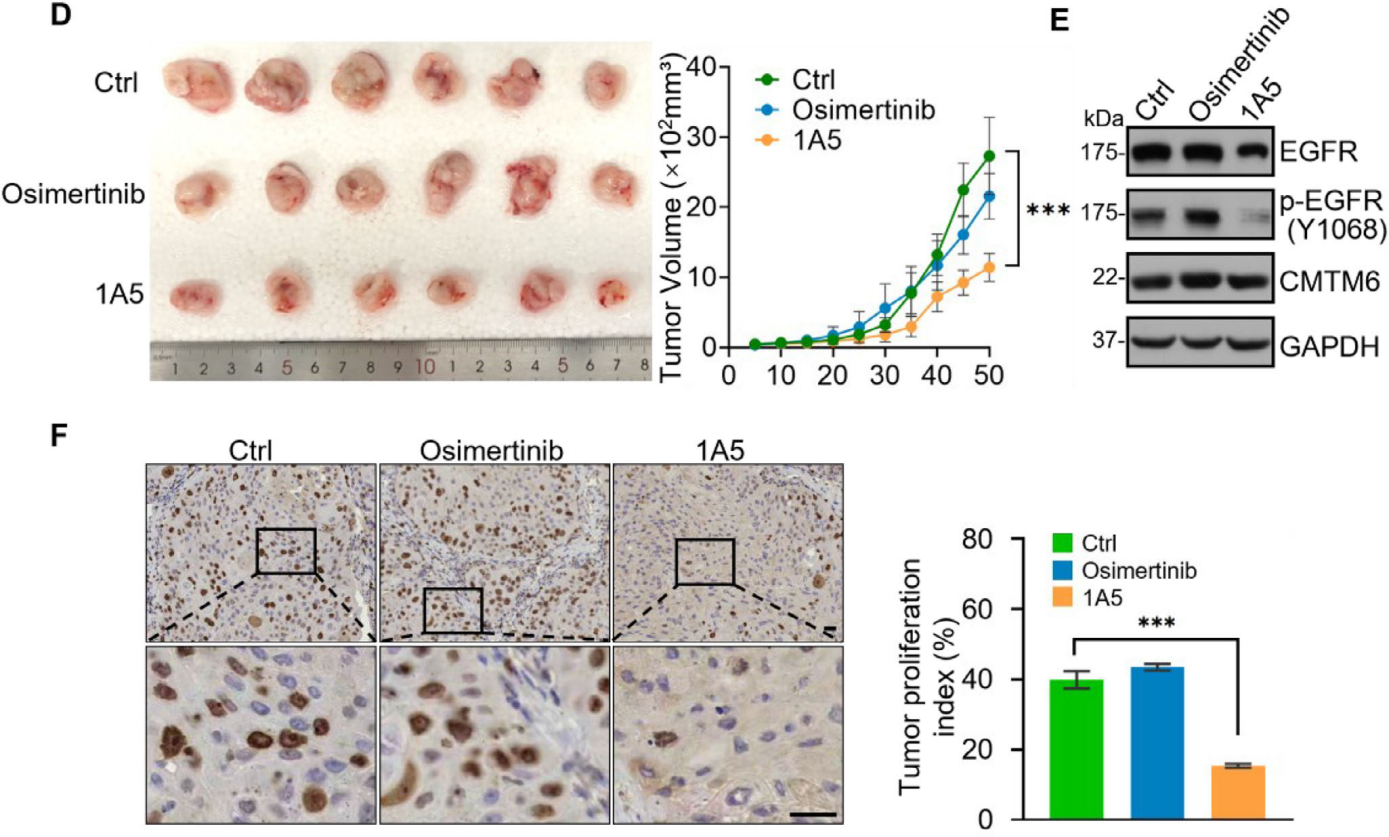

A PDX model was established from an advanced‐stage EGFR‐mutated (L858R+T790M+MET amplification) lung adenocarcinoma patient that had progressed following resistance to both 2nd generation TKI (afatinib) and 3rd generation TKI (osimertinib). The patient tumor tissues were subcutaneously implanted into 6‐week‐old female NCG mice (*n* = 6), with 1A5 subcutaneously injected seven times that 1A5 treatment rendered a significantly inhibited growth of the patient tumor (Figure 6D), an effect that was not evident when osimertinib treatment was applied via gavage once daily for two weeks, as expected due to the tumor's TKI‐resistant nature (Figure 6D, and Figure S1B, Supporting Information). Immunoblotting with antibodies against EGFR and CMTM6 of the lysates from the PDX tumors treated with control, osimertinib, or the nanobody 1A5 revealed that 1A5 treatment was associated with a significantly reduced level of EGFR (Figure 6E). Immunostaining of the PDX tumor sections or Ki‐67 showed that 1A5 treatment led to a significant reduction in cell proliferation, which was not observed in osimertinib‐treated tumors (Figure 6F). Collectively, these results demonstrated an inhibitory effect of the CMTM6‐targeting nanobody 1A5 on the growth of TKI‐resistant NSCLC tumors, supporting the therapeutic potential of 1A5 in NSCLC intervention to overcome the resistance to EGFR TKIs.

## 3 Discussion

The emergence of drug resistance to EGFR TKIs remains a major obstacle to the successful treatment of relevant malignancies including NSCLC, often leading to poor patient prognosis.^[^ ^8a^ ^]^ In the current study, we identified that CMTM6 acts as a stabilizer of EGFR in endocytic trafficking. We showed that the level of CMTM6 was elevated in NSCLC tumors, and high expression of CMTM6 was associated with a poor prognosis of the patients in a TCGA NSCLC cohort. We explored the therapeutic potential of a CMTM6‐targeting nanobody that we developed for the treatment of EGFR TKI‐resistant NSCLC.

Multifaceted strategies have been applied to address TKI‐resistance in NSCLC, encompassing next‐generation EGFR inhibitors, combination therapies targeting alternative pathways like MET and MEK, and exploration of novel therapeutic targets such as HER2 and BRAF, along with combined immunotherapy.^[^ ^31^ ^]^ Functional genomics and proteomics studies have identified several protein factors including TRIB3, SGCE, and SERPINE2 as EGFR interactors that prevent EGFR from ubiquitination and degradation,^[^ ^32^ ^]^ suggesting that blocking the interaction of these factors with EGFR could represent another strategy to potentially destabilize EGFR and inhibit EGFR oncogenic signaling. Additionally, factors that are functionally linked to protein trafficking and sorting, such as PGK1, ARL4A, and LAPTM4B, have been found to interact with EGFR to regulate the trafficking and degradation of EGFR, making them yet another potential target to modulate EGFR stability and signaling.^[^ ^33^ ^]^ However, EGFR protein and its cellular signaling appear to be intricate and complicated. For example, EGFR mutants in NSCLC exhibit constitutive internalization and are resistant to degradation due to impaired interaction with E3 ubiquitin ligase CBL, thus leading to prolonged oncogenic signaling.^[^ ^34^, ^35^ ^]^ In the present study, we found that CMTM6 is physically associated and co‐localized with EGFR mutants at the cell membrane and in recycling endosomes that are marked by RAB11, thereby promoting lysosome‐mediated degradation of EGFR. It remains to be explored whether EGFR mutants bypass early endosomes and are routed directly to RAB11‐positive recycling endosomes and whether the CMTM6‐EGFR complex involves intermediary proteins, such as myosin V, actin filaments, and the exocyst complex, which are known to facilitate the docking of cargo like EGFR at the plasma membrane.^[^ ^36^ ^]^ Given our findings that CMTM6 is overexpressed in NSCLC tumors where the colocalization of CMTM6, EGFR, and RAB11 is enhanced, and in light of the observation that high expression of CMTM6 is associated with a poor prognosis in NSCLC patients, our study highlights the role of CMTM6 in mediating endocytic trafficking of tumor‐associated EGFR and underscore its potential implications in clinical intervention of NSCLC.

CMTM6 has been previously reported to regulate the stability of multiple membrane proteins. It has been reported that CMTM6 interacts with PD‐L1 at the cell membrane and in recycling endosomes, preventing PD‐L1 from lysosomal degradation and thereby inhibiting tumor‐specific T‐cell activity.^[^ ^14^, ^15^ ^]^ We reported previously that the anti‐CMTM6 nanobody 1A5 reduces T‐cell immunosuppression and enhances the apoptotic susceptibility of tumor cells by interfering with PD‐1/PD‐L1 signaling.^[^ ^30^ ^]^ CMTM6 has also been reported to stabilize the HER2 protein, which drives the development of trastuzumab resistance in HER2‐positive breast cancer by inhibiting HER2 ubiquitination and stabilizing cell surface CD58 from lysosome‐mediated degradation.^[^ ^14a^ ^]^ In the current study, we showed that the CMTM6‐targeting nanobody blocks the CMTM6‐EGFR interaction, inhibits TKI‐resistant NSCLC proliferation, and suppresses the growth of EGFR‐TKI‐resistant NSCLC. Beyond our work with the CDX model, we also evaluated the inhibitory effects of the nanobody on tumor growth using PDX models representing a patient that had developed resistance to both 2nd and 3rd generation EGFR‐TKI agents. Advantageous features of nanobodies that make them particularly suitable for tumor treatment include their small size, high affinity, target specificity, thermal stability, reversible folding, compatibility with genetic engineering methods, and excellent tissue penetration. In addition to nanobodies, other nanoscale compounds, such as a copper (II) agent based on human serum albumin and a rhodium (III) complex delivered via apoferritin,^[^ ^37^ ^]^ have been shown promise in overcoming drug resistance of NSCLC. Further studies will be needed to evaluate the clinical potential of these nanomaterials in the treatment of NSCLC.

Moreover, beyond NSCLC, aberrant EGFR signaling also manifests in colorectal cancer and head and neck cancer.^[^ ^38^ ^]^ Patients with EGFR overexpression in colorectal and head and neck cancers are commonly treated with the EGFR monoclonal antibody cetuximab. Concurrently, immune checkpoint inhibitors, particularly PD‐1/PD‐L1 inhibitors, have demonstrated efficacy in restraining tumor progression in subsets of colorectal and head and neck cancer patients with positive PD‐L1 expression.^[^ ^39^ ^]^ Therefore, we proposed that patients exhibiting dual features—EGFR overexpression and positive PD‐L1 expression—may derive substantial benefit from CMTM6 targeting nanobodies, and the therapeutic efficacy of the CMTM6 nanobody could potentially surpass that of monotherapy with either EGFR or PD‐L1 inhibitors. By strategically targeting CMTM6, the complex interplay between EGFR signaling, drug resistance, and immune evasion pathways could be disrupted, potentially providing a new avenue for treatment. However, the effect of the CMTM6 targeting nanobody on colorectal cancer and head and neck cancer still needs to be investigated and the impact of this nanobody on the immune microenvironment remains to be studied.

In summary, our study reports that CMTM6 interacts with and stabilizes mutant EGFR via recycling endosomes. We developed an anti‐CMTM6 nanobody that blocks CMTM6‐EGFR interaction, reducing EGFR protein level and overcoming EGFR‐TKI resistance in NSCLC. We identify CMTM6 as a stabilizer of mutant‐EGFR, supporting targeting CMTM6 as a promising therapeutic strategy for NSCLC.

## 4 Experimental Section

### Cell Lines and Cell Culture

PC‐9, A549, and H1299 cells obtained from ATCC were cultured in DMEM medium with 10% FBS, 100 U mL^−1^ penicillin, and 100 mg mL^−1^ streptomycin at 37 °C with 5% CO_2_. NCI‐H1975 and HCC827 cells from ATCC were cultured in RPMI‐1640 medium with 10% FBS under similar conditions. PC‐9‐gefitinib resistant (GR) and NCI‐H1975‐osimertinib resistant (OR) cells were generated by gradually increasing gefitinib or osimertinib concentrations, starting from 500 nM and increasing by 500 nM every 15 days until reaching a final concentration of 2 µM. Single clones were selected and maintained in a culture medium containing 2 µM of gefitinib or osimertinib. The acquired mutations were verified by next‐generation sequencing.

### Mice

Six‐week‐old female BALB/c nude mice were procured from Peking University Health Science Center for CDX experiments. Six‐week‐old female NCG mice were procured from Gempharmatech Inc (Nanjing, China) for PDX experiments. All animal procedures were conducted in accordance with the guidelines of the Institutional Animal Care and Use Committee of Peking University Health Science Center (Approval No. LA2020044).

### Plasmids and Small Interfering RNA Transfection

The cDNA sequences of CMTM6 and EGFR were amplified by PCR and ligated into pcDNA3.1 vector that contains three copies of FLAG or pGEX‐4T‐3 vector. Mutants of EGFR including EGFR/L858R, /T790M, and /L858R+T790M were generated using QuikChange Lighting Site‐Directed Mutagenesis Kit. All clones were confirmed by DNA sequencing. Transfections of these plasmids were performed using polyethyleneimine (PEI) as per the manufacturer's instructions. siRNAs targeting CMTM6 were designed, and synthesized by GenePharma Inc (Shanghai, China), and transfected into cells at a final concentration of 20 nM using RNAiMAX reagent. The sequences of the siRNAs were as follows: siCMTM6‐1: 5′‐GGCGCGUUCUCAAGGGCUUTT‐3′, siCMTM6‐2: 5′‐GCUGCAAUUGUGUUUGGAUTT‐3′.

### Reagents

CHAPS, anti‐FLAG M2 affinity gel, and FLAG peptide (Sigma), phosphatase inhibitor (Applygen), protease inhibitor cocktail (Roche Applied Science), Protein A/G Sepharose CL‐4B beads (Amersham Biosciences), NuPAGE 4%–12% Bis‐Tris gel (Invitrogen), and silver‐stained kit (Pierce).

### Immunopurification and Mass Spectrometry

HEK293T cells stably expressing FLAG‐tagged EGFR/L858R were washed twice with cold phosphate‐buffered saline (PBS), scraped, and collected by centrifugation at 800 × g for 5 min. Cellular extracts were prepared by lysing the cells in CHAPS buffer (1% CHAPS, 50 mM Tris‐HCl pH 7.5, 150 mM NaCl) containing a protease inhibitor cocktail (Roche). Anti‐FLAG immunoaffinity resin (Sigma) was prepared according to the manufacturer's protocol. Cell lysates were applied to the immunoaffinity resin to enable adsorption of the protein complex. After binding, the resin was washed with CHAPS buffer. FLAG peptide (Sigma) was applied to the resin to elute the FLAG‐tagged protein‐associated complex as described by the vendor. The eluates were collected and resolved on NuPAGE 4%–12% Bis‐Tris gel (Invitrogen), silver‐stained (Pierce), and subjected to LC‐MS/MS for sequencing and data interpretation.

### Immunoprecipitation and Immunoblotting

Cell lysates were prepared by incubating the cells in CHAPS buffer containing protease inhibitor cocktail (Roche) for 40 min at 4 °C, followed by centrifugation at 14000 × g for 15 min. For immunoprecipitation, 750 mg of protein lysates was incubated with 2 µg of antibodies for 12 h at 4 °C with constant rotation, followed by the addition of 50 µL of 50% protein A/G agarose beads (Amersham Biosciences) and incubation for another 2 h. Beads were then washed five times using the CHAPS buffer. The precipitated proteins were eluted from the beads by resuspending the beads in 2 × SDS‐PAGE loading buffer and boiling for 10 min. The resultant materials from immunoprecipitation or cell lysates were resolved using SDS‐PAGE gels and transferred onto nitrocellulose membranes. For immunoblotting, membranes were incubated with appropriate antibodies overnight at 4 °C followed by incubation with a secondary antibody for 2 h at room temperature. Immunoreactive bands were visualized using immunoblotting Luminol reagent (Santa Cruz) according to the manufacturer's recommendation. The following antibodies were used: anti‐FLAG (Sigma), anti‐EGFR and anti‐Phospho‐EGFR (Tyr1173) (Abclonal), anti‐CMTM6 and anti‐Phospho‐EGFR (Tyr1068) (Cell Signaling Technology), anti‐RAB7 (Abcam), anti‐GAPDH (TransGen Biotech), anti‐HA (MBL), and anti‐RAB11 (BD Transduction Laboratories).

### Proximity Ligation Assay (PLA)

Proximity ligation assay was performed using the Duolink PLA Fluorescence Kit (Sigma) according to the manufacturer's instructions. PC‐9 cell slides were fixed in 100% methanol at −20 °C for 10 min. After incubation with Duolink blocking solution at 37 °C for 1 h, slides were treated with anti‐EGFR (Abcam) and anti‐CMTM6 (Cell Signaling Technology) primary antibodies overnight at 4 °C. Subsequently, slides were incubated with proximity ligation assay probes PLUS and MINUS for 1 h at 37 °C, followed by ligation mix solution for 30 min at 37 °C. After ligation, slides were incubated with an amplification polymerase solution for 100 min at 37 °C. Nuclei were counterstained with DAPI. Images were captured using a confocal fluorescent microscope.

### Flow Cytometry

Cells were washed and resuspended in PBS containing 2% FBS and 0.09% sodium azide (NaN3) and stained anti‐EGFR with Allophycocyanin (APC) (BioLegend). Subsequently, cells were subjected to flow cytometry analysis, and the results were analyzed using FlowJo.

### Immunofluorescent Staining

PC‐9 cells in 6‐well culture slides were fixed in 100% methanol for 10 min at −20 °C. For double immunofluorescence staining, cells were blocked with 4% FBS for 1 h and then stained with specific primary antibodies, followed by corresponding secondary antibodies. Nuclei were counterstained with DAPI, and images were captured using a confocal fluorescent microscope. For multicolor immunofluorescence staining, the PANO 4‐plex TSA kit (Panovue) was utilized according to the manufacturer's instructions. Three antibodies were sequentially applied, followed by horseradish peroxidase‐conjugated secondary antibody incubation and tyramide signal amplification. Signal amplification was performed with tyramide reagents conjugated to fluorophores. The staining procedure was conducted in consecutive rounds, with each round consisting of blocking, primary antibody, secondary HRP‐labeled antibody, tyramide reagents, and antibodies elution. A special stripping buffer (Panovue) was used for antigen retrieval after each tyramide signal amplification operation. Nuclei were stained with DAPI after labeling all the antigens, and images were quantified using Olympus FV31S‐SW and ImageJ software. The following antibodies were used: anti‐CMTM6 (Atlas), anti‐RAB5 (Santa Cruz Biotechnology), anti‐EGFR and anti‐LAMP1 (Cell Signaling Technology), anti‐EEA1 (Invitrogen), anti‐RAB7 and anti‐EGFR (Abcam), and anti‐RAB11 (BD Transduction Laboratories).

### Nanobody Production and Screening

The generation of the nanobodies targeting CMTM6 was performed essentially the same as previously described.^[^ ^30^ ^]^ Briefly, a *Camelus bactrianus* was immunized subcutaneously seven times with ≈10^8^ HEK293S cells expressing the target protein. Peripheral blood lymphocytes (200 mL) were collected 3–5 days after the final immunization. A nanobody phage library (∼10^8^ transformants) was then constructed following standard protocols. For nanobody selection, nanobody‐displaying phages were rescued by infecting the library (OD600 0.4–0.5) with 20‐fold excess helper phage at 37 °C. After centrifugation, the pellet was resuspended in 2YT medium with ampicillin (100 µg mL^−1^) and kanamycin (25 µg mL^−1^) and cultured overnight (37 °C, 200 rpm). Phages were precipitated with 10% (w/v) PEG3000/1.25 M NaCl, resuspended in ice‐cold PBS, and further purified. MaxiSorp plates coated with neutravidin (10 µg mL^−1^) were blocked (2% milk/PBS, 2 h, room temperature), and biotin‐labeled target protein was immobilized. Phages were then incubated (30 min, 4 °C), washed extensively with PBS, and eluted using 0.25 mg mL^−1^ trypsin (30 min, room temperature). Eluted phages infected exponential‐phase TG1 cells and were plated to enrich for target‐specific clones over multiple selection rounds. Individual TG1 colonies were grown in 2YT, induced with 1 mM IPTG, and periplasmic nanobody extracts were screened by ELISA.

### Nanobody Expression, Purification

Plasmids from final positive clones were transformed into *Top10F′* cells and plated on LB agar containing ampicillin (100 µg mL^−1^). A single colony was cultured overnight, then expanded in 1 L of medium at 37 °C (220 rpm) until OD600 reached 0.6–0.8. After inducing protein expression with 1 mM IPTG (overnight, 28 °C, 220 rpm), cells were harvested (4500 rpm, 15 min, room temperature), lysed by ultrasonication, and loaded onto a nickel affinity column. The flow‐through containing nanobody was further purified using a HiLoad 16/600 Superdex 75 column.

### Cell Viability/Proliferation Assay

The PC‐9‐GR and NCI‐H1975‐OR cells were plated in 96‐well plates with equal medium volumes, and either Ctrl (DMSO for EGFR‐TKI and IgG for 1A5), EGFR‐TKI, or 1A5 (10 µg mL^−1^) was added to PC‐9‐GR and NCI‐H1975‐OR cells every 24 h. On the day of harvest, the TransDetect Cell Counting Kit (CCK) (TransGen Biotech) was used according to the manufacturer's protocol. Plates were incubated at 37 °C for 2 h, and cell viability was determined by measuring the absorbance of the converted dye at a 450 nm wavelength, following the manufacturer's protocol. Each experiment was conducted in triplicate and repeated at least three times.

### Colony Formation Assay

PC‐9‐GR or NCI‐H1975‐OR cells were cultured in media in 96‐well plates for 14 days. Either Ctrl (DMSO for EGFR‐TKI and IgG for 1A5), EGFR‐TKI, or 1A5 (10 µg mL^−1^) was added every 24 h. Following incubation, cells were fixed with 4% paraformaldehyde, stained with 0.1% crystal violet for colony visualization, and counted using a light microscope. Each experiment was performed in triplicate and repeated at last three times.

### Tumor Xenografts

For cell‐derived xenografts (CDX) models, PC‐9‐GR or NCI‐H1975‐OR cells (5–8 × 10^6^ cells) were injected subcutaneously into six‐week‐old female BALB/c nude mice. According to body equal completely randomized design, mice were divided into groups of six before injection. After one week, gefitinib or osimertinib was administered by gavage daily for two weeks at 30 mg k^−1^g, while hIgG1 or 1A5‐Fc was given intraperitoneally for a total of seven doses. hIgG1 was administered at a dose of 200 µg/each, while 1A5‐Fc was administered at a dose of 200 µg/each. The control agents for EGFR‐TKI, carboxymethyl cellulose sodium (CMC‐Na), and for 1A5, immunoglobulin G (IgG), were administered in a manner corresponding to each respective drug. Tumor size was measured daily using a vernier caliper, and volume was calculated accordingly. For patient‐derived xenografts (PDX) models, tumor tissues were obtained from the West China Hospital of Sichuan University. Written informed consent was obtained from all participants, and the protocol was approved by the Ethics Committee on Biomedical Research of West China Hospital of Sichuan University (Approval No. 2020353). Tissues from an advanced‐stage EGFR‐mutant (L858R/T790M) lung adenocarcinoma patient, resistant to both afatinib and osimertinib, were implanted subcutaneously into 6‐week‐old female NCG mice (*n* = 6). Osimertinib was administered by gavage for two weeks, and 1A5 was injected intraperitoneally at 80 mg k^−1^g for a total of seven doses. Tumors were measured every 5 days. PDX tumors were collected for immunoblotting analysis and immunohistochemical staining. All studies were approved by the Ethics Committee of the Peking University Health Science Center (Approval No. PUIRB‐2021272).

### Tissue Immunohistochemistry

Primary tumors from female BALB/c nude or NCG mice were collected and fixed with 10% neutral buffered formalin for 24 h. Antigen retrieval was performed using high pressure and incubation in 0.01 m sodium citrate buffer. The samples were then blocked with 10% normal goat serum in PBS and incubated overnight at 4 °C with primary antibodies. After washing with PBS, the samples were incubated with polymer HRP goat anti‐rabbit (Dako, Agilent) for 30 min at room temperature, followed by development with DAB (3,3′‐diaminobenzide tetrahydrochloride) and counterstaining with hematoxylin (Zhongshan Golden Bridge Biotechnology Company). Evaluation of all specimens was conducted by two pathologists blinded to the experimental groups. The following antibodies were used: anti‐CMTM6, anti‐EGFR (Cell Signaling Technology), anti‐RAB7 (Abcam), anti‐PanCK (Gene tech), and anti‐RAB11 (BD Transduction Laboratories).

### Multiplexed Immunohistochemistry (mIHC) and Imaging Analysis

A lung cancer tissue array (HLugA180Su08) was procured from Shanghai Outdo Biotech Company. Informed consent was obtained from all patients prior to the research. mIHC was conducted using an Opal 6‐color fluorescent IHC kit (PerkinElmer) combined with automated quantitative analysis (AQUA; Genoptix) to evaluate the expression of CMTM6, EGFR, PanCK, and RAB11. After optimizing antibody concentration and sequence, a spectral library based on single‐stained slides was established. Following dewaxing and rehydration, slides underwent heat‐induced antigen retrieval before mIHC staining. Secondary antibodies included anti‐mouse Envision HRP (Dako) and anti‐rabbit Envision HRP (Dako). Imaging was performed using the Vectra Quantitative Pathology Imaging System (PerkinElmer), with spectral unmixing applied to distinguish fluorescence signals. Subsequently, InForm (PerkinElmer) image analysis was conducted, incorporating tissue segmentation, cell segmentation, and phenotyping.

### Statistical Analysis

All data were inspected for outliers and assessed for normality and homogeneity of variances via Levene's test prior to analysis. If necessary, data that did not meet assumptions of normality were either transformed or analyzed using appropriate nonparametric methods. Unless otherwise noted, results are presented as the mean ± standard deviation (SD) or mean ± standard error of the mean (SEM). Sample sizes for each experiment and statistical test are provided in the figure legends. Comparisons between the two groups were performed using a two‐tailed Student's t‐test (paired or unpaired, as appropriate) when normality and variance assumptions were met. For comparisons among three or more groups, a one‐way analysis of variance (ANOVA) was conducted, followed by Tukey's HSD test to correct for multiple comparisons. For all tests, the significance level was set at 0.05. Statistical significance is indicated in the figures as *p < 0.05, **p < 0.01, ***p < 0.001. All statistical analyses were performed using SPSS version 19.0. mIHC data were analyzed using R programming.

## Conflict of Interest

The authors declare no conflict of interest.

## Supporting information



Supporting Information

## Data Availability

The data that support the findings of this study are available from the corresponding author upon reasonable request.
