# Supplementary material for: A CMTM6 Nanobody Overcomes EGFR‐TKI Resistance in Non‐Small Cell Lung Cancer
Source: Adv Sci (Weinh). 2025 Jun 16;12(27):2410945. doi: 10.1002/advs.202410945 (PMC12279249; doi:10.1002/advs.202410945)

Supporting Information for

**A CMTM6 Nanobody Overcomes EGFR-TKI Resistance in Non-small Cell Lung Cancer**

*Lu Xia, Jichuan Wang, Hui Xue, Haimeng Li, Qinghua Li, Sen Qin, Chunyu Yu, Yanhua Liu, Yu Gao, Lingyun Li, Sudun Guan, Enrun Zheng, Feiya Suo, Lin He, Yongsheng Wang, Wenling Han*, Yongfeng Shang*, Yong Geng*, and Luyang Sun**

**Figure S1**


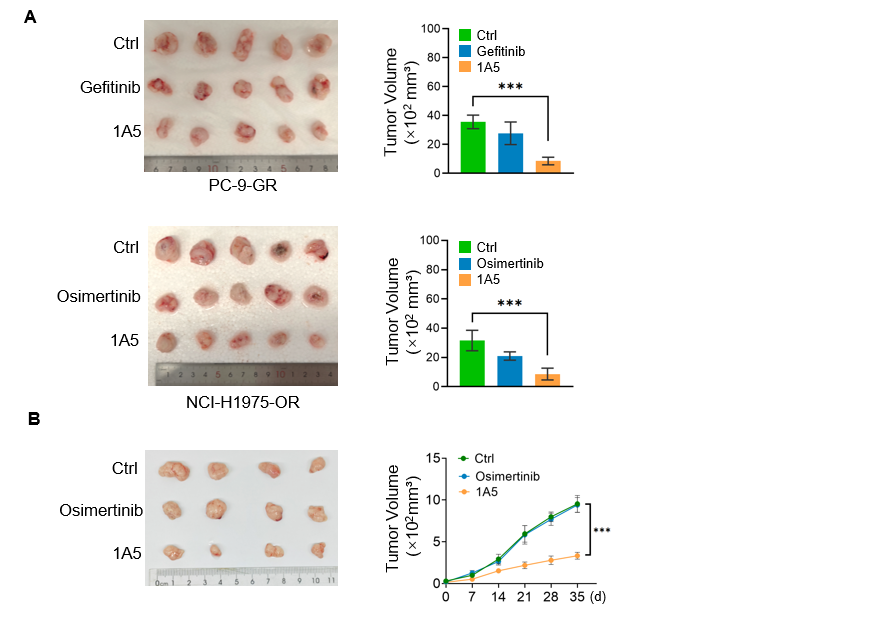


**Figure S1**. Anti-CMTM6 Nanobody Suppresses the Growth of EGFR-TKI-Resistant NSCLC Tumor *in Vivo*. A) PC-9-GR or NCI-H1975-OR cells were subcutaneously injected into 6-week-old female BALB/c nude mice (n = 5), respectively. Gefitinib or osimertinib was administered by gavage daily for two weeks, and hIgG1 or 1A5-Fc was administered intraperitoneally for seven doses. Tumor size was measured at 8 weeks post-injection. Representative images of primary tumors are shown (****p* < 0.001). B) Patient-derived tumor tissues were subcutaneously implanted into 6-week-old female NCG mice (n = 4). Osimertinib was administered daily by gavage for two weeks, and hIgG1 or 1A5-Fc was administered intraperitoneally for seven doses. Tumor size was measured at 35 days post-implantation. Representative images of primary tumors are shown (****p* < 0.001).

**Figure S2. Uncropped blots for all the western blots in this study.**


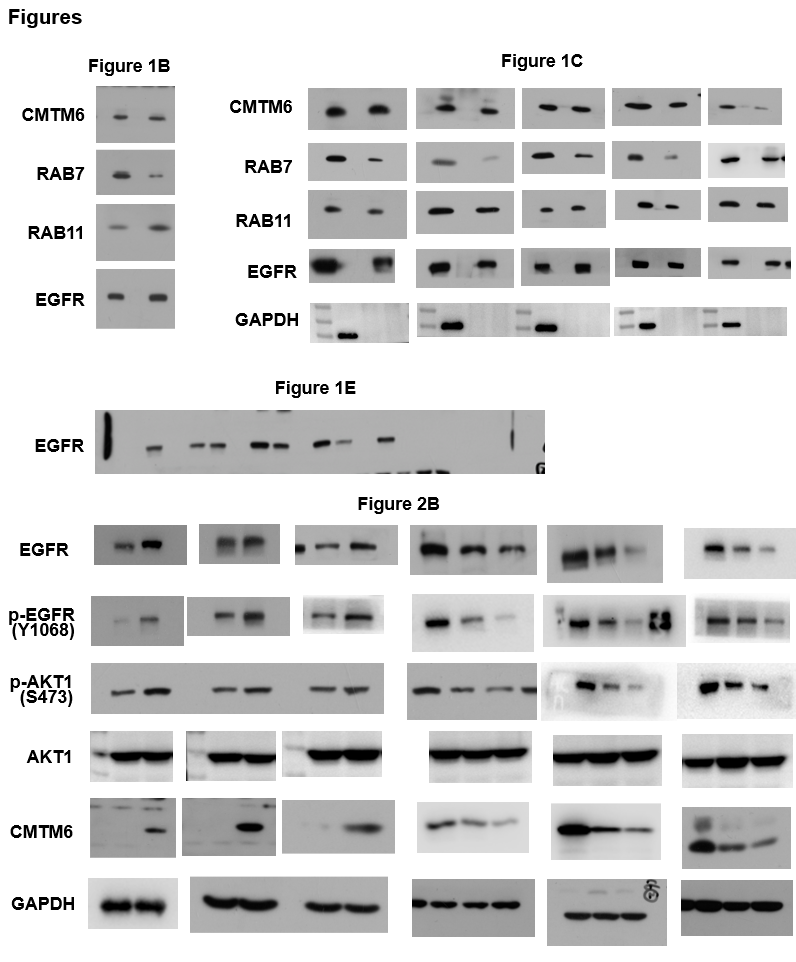


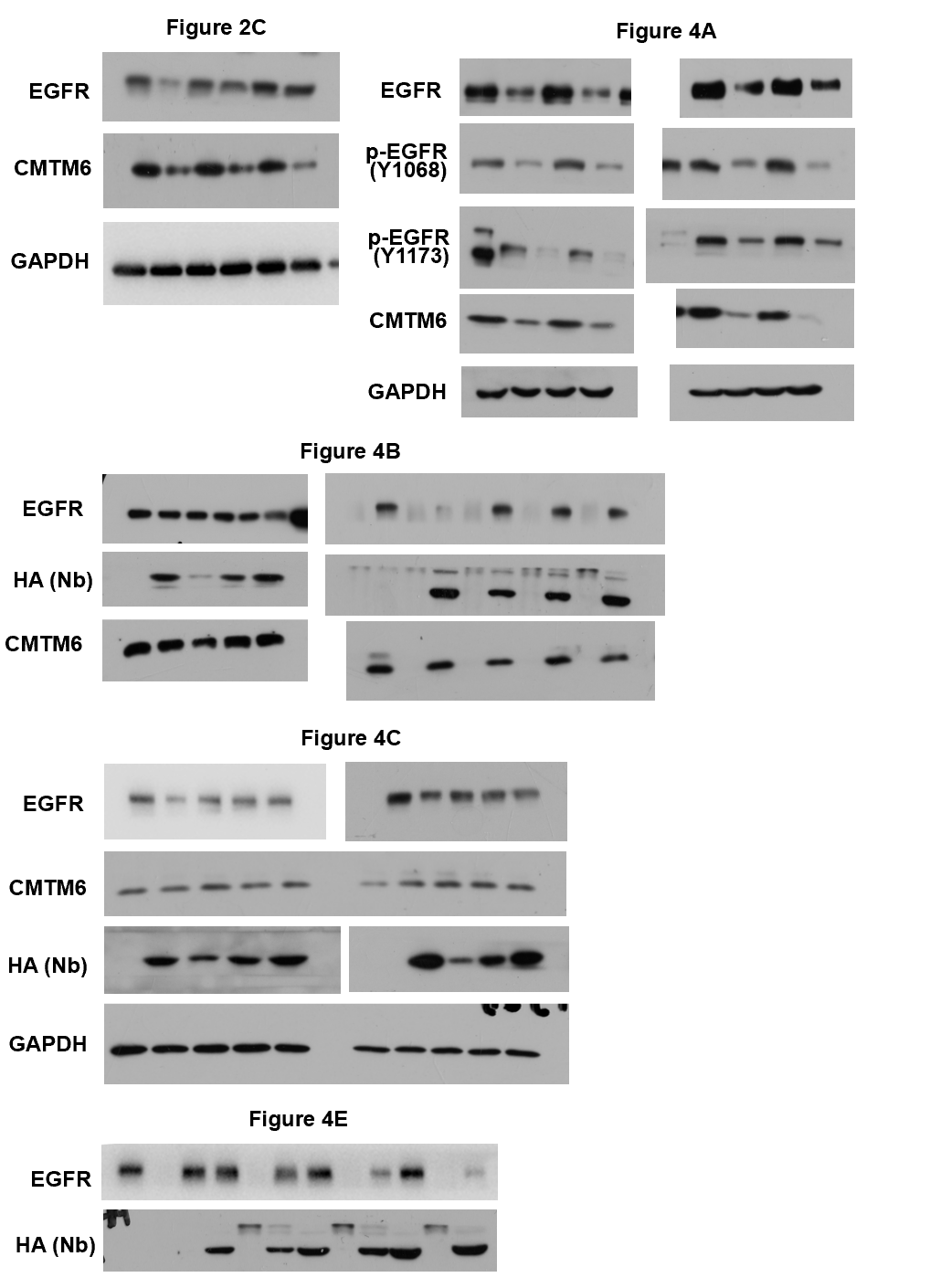


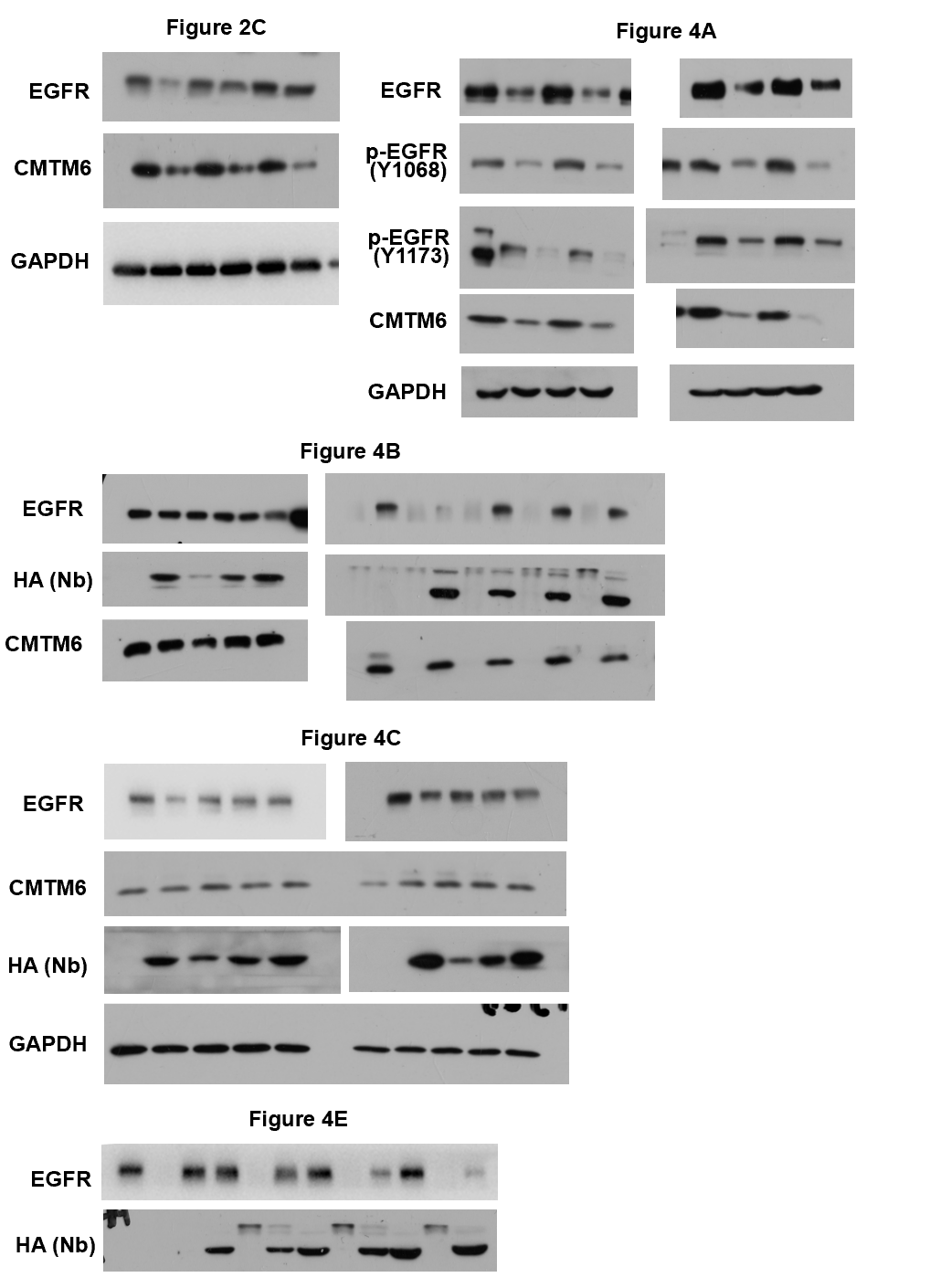


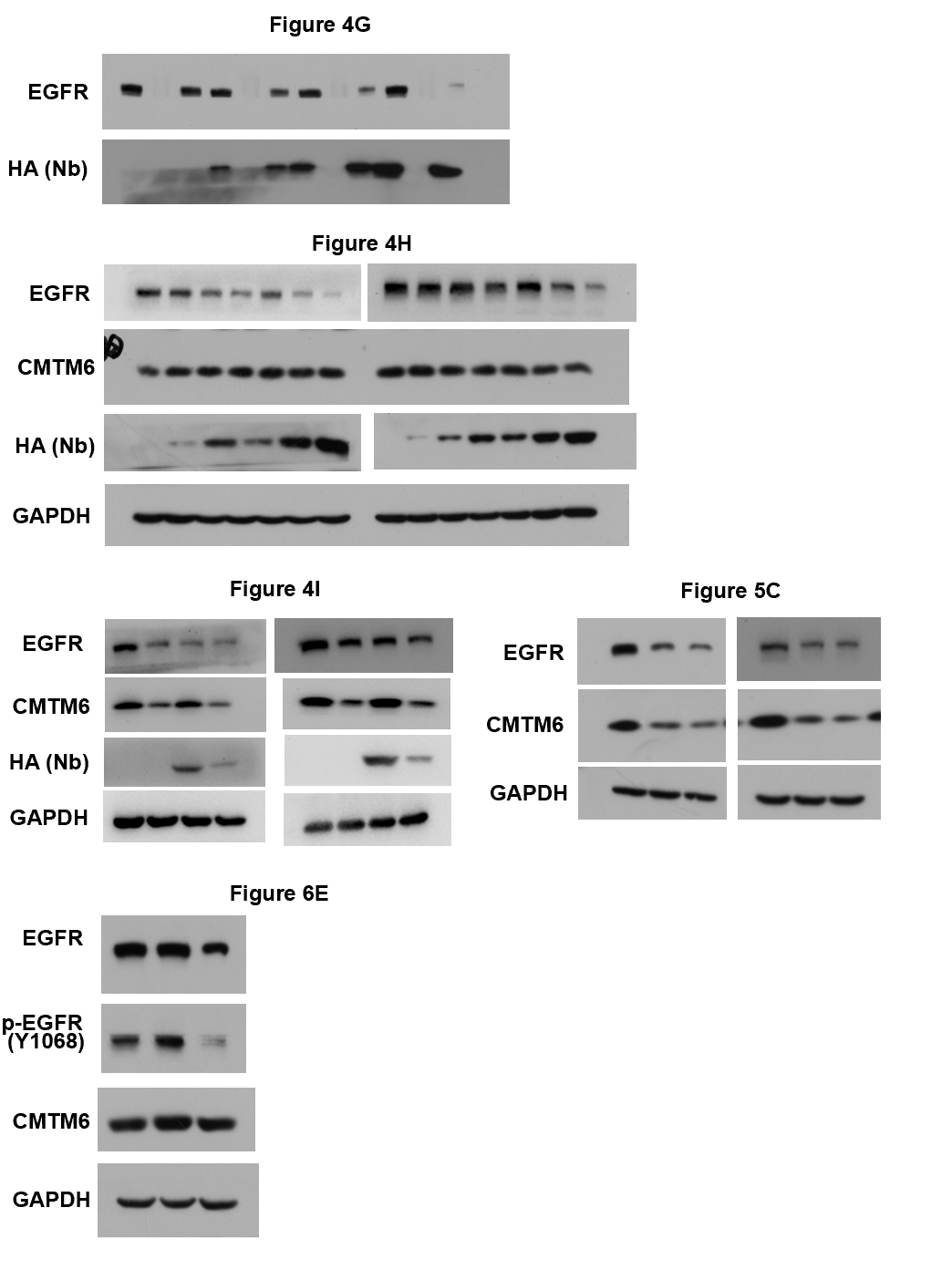


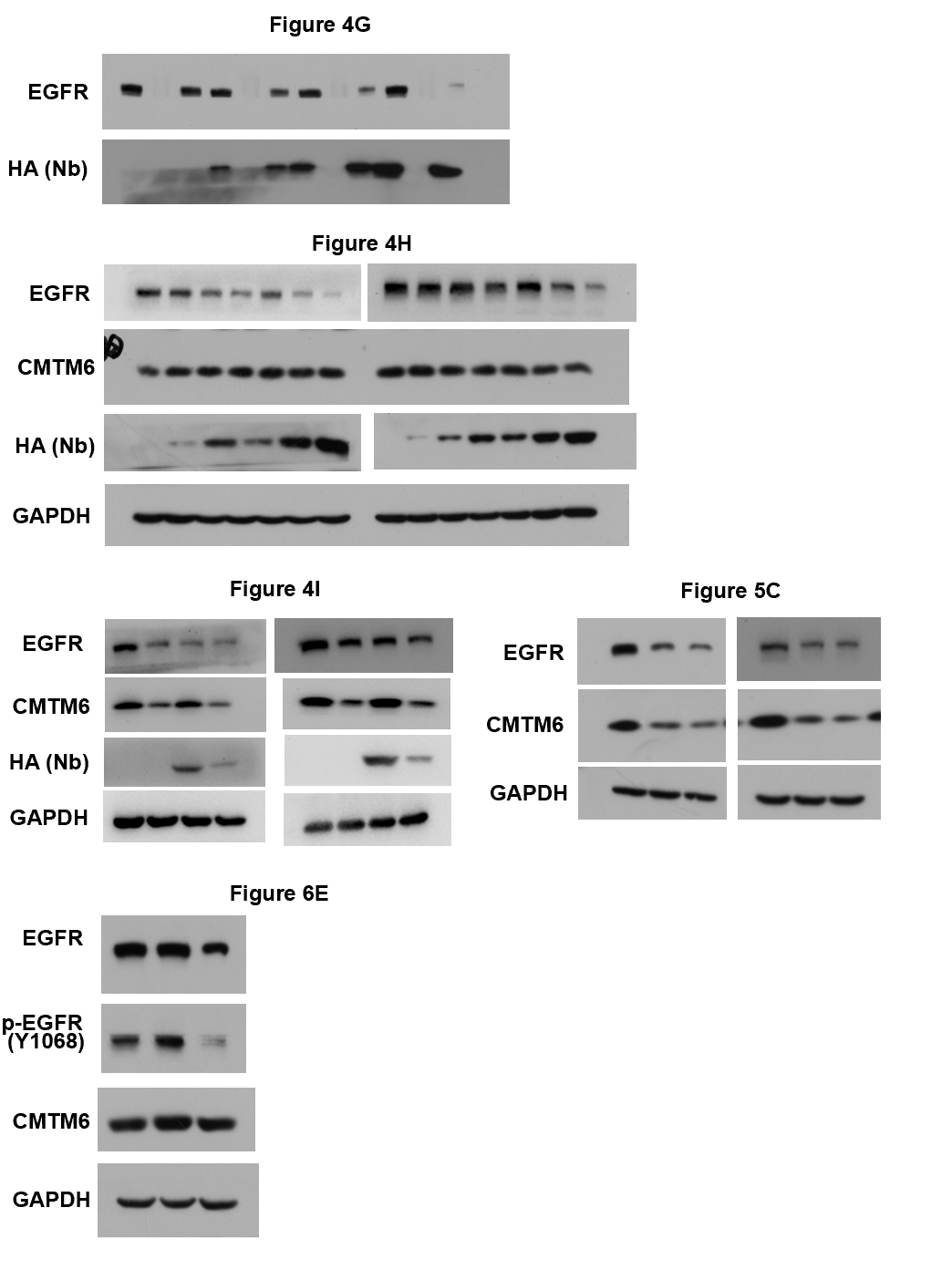


**Figure S3. Uncropped microscope images in this study.**

**Figure 1D**


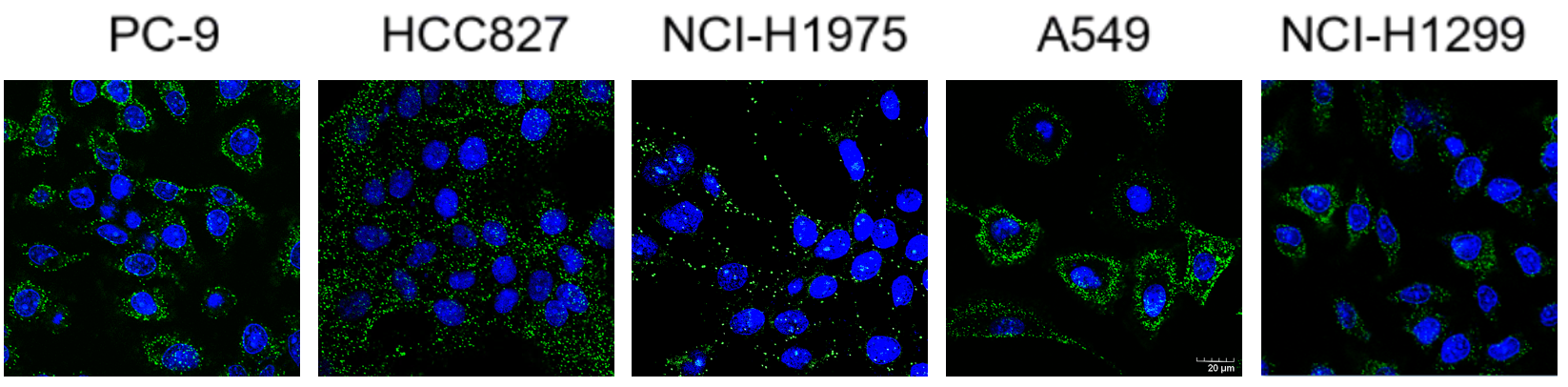


**Figure 2D**


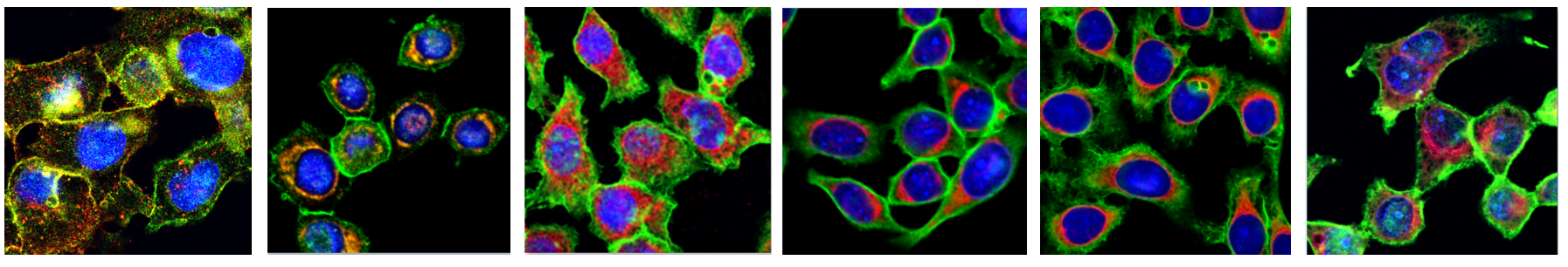


**Figure 2E**


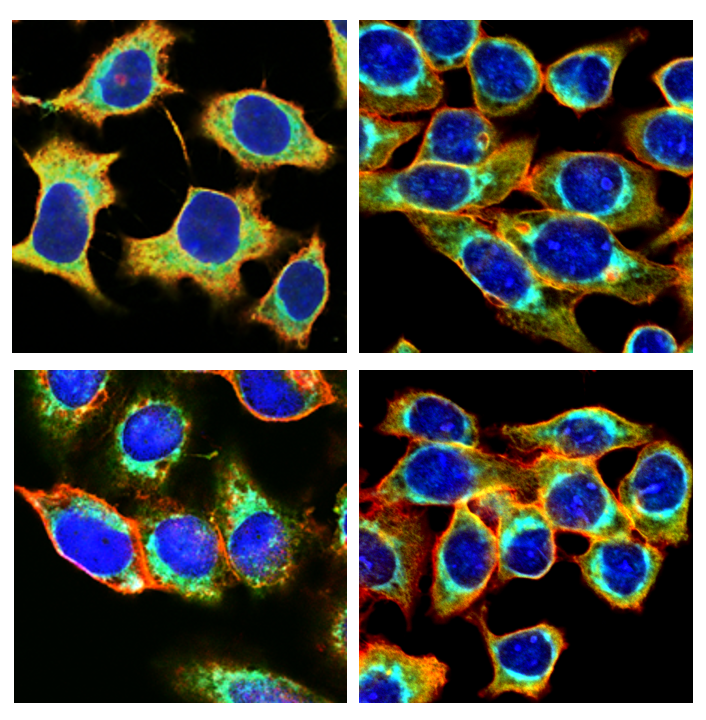


**Figure 3B**


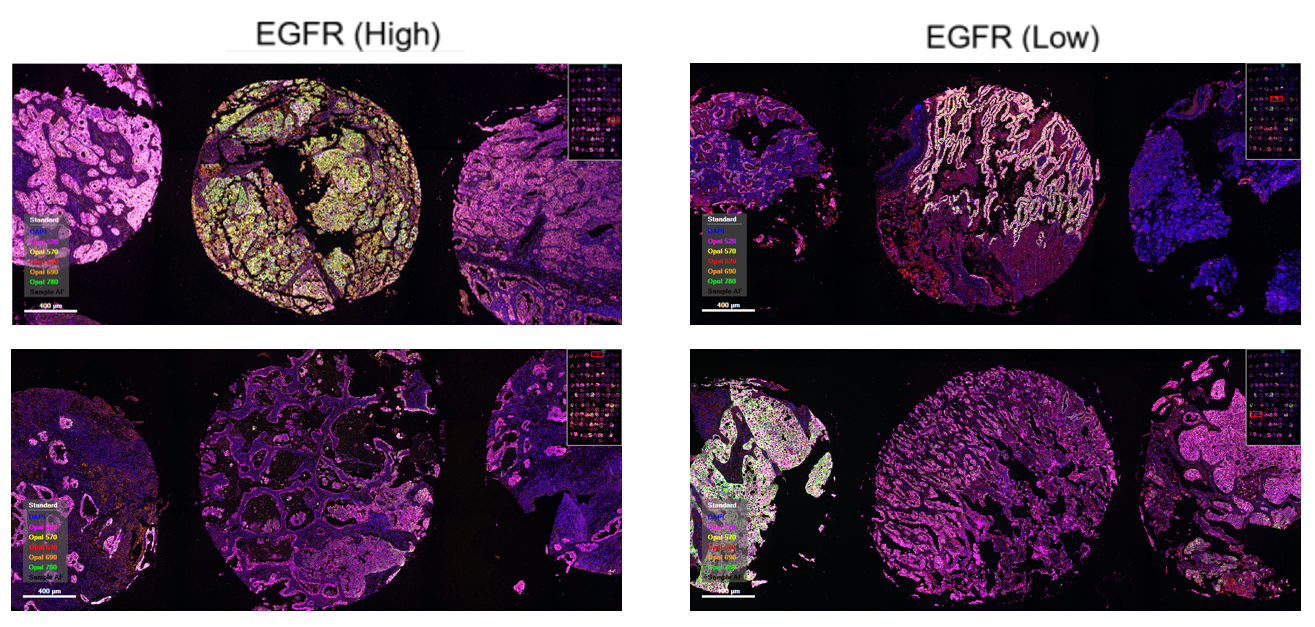


**Figure 4D**


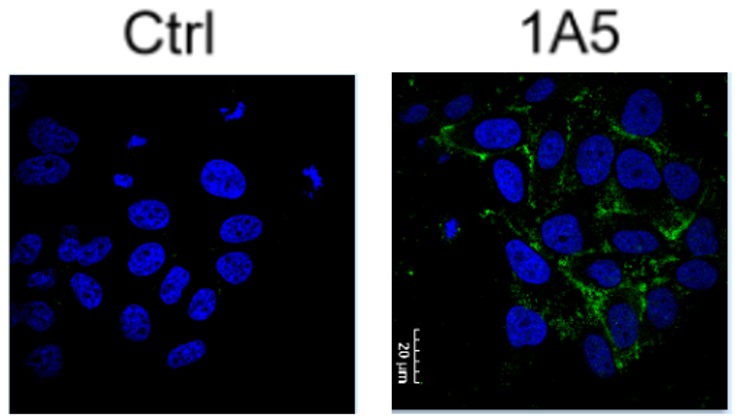


**Figure 4F**


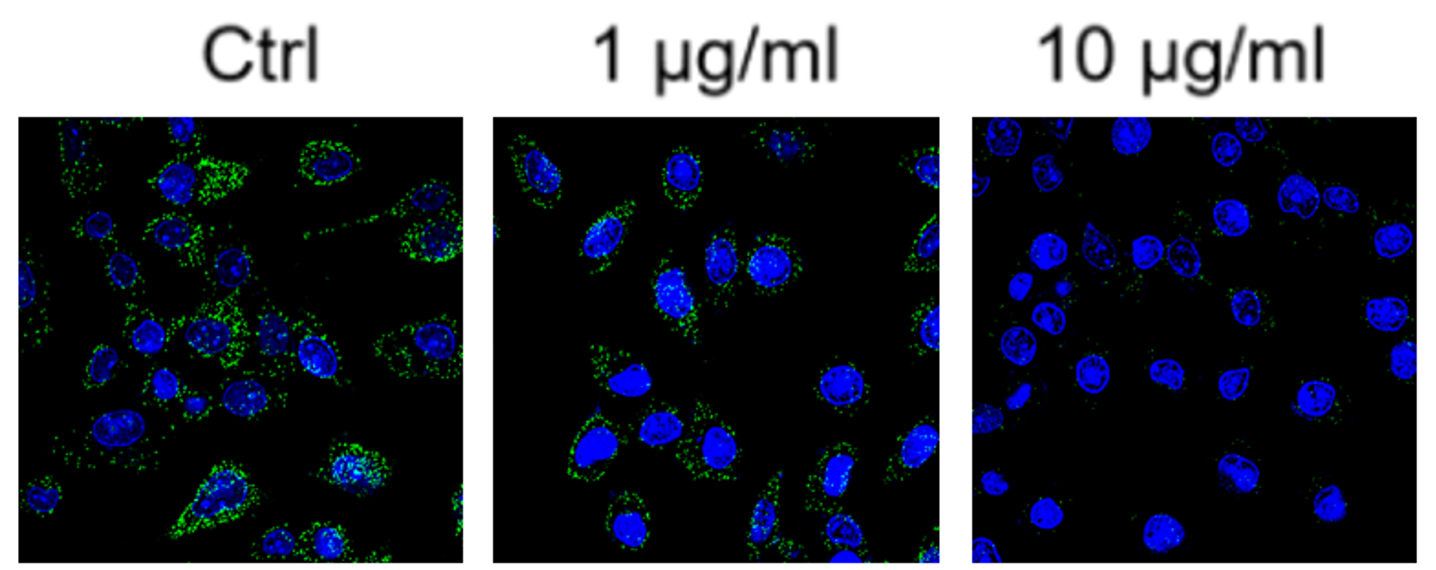


**Figure 6C**


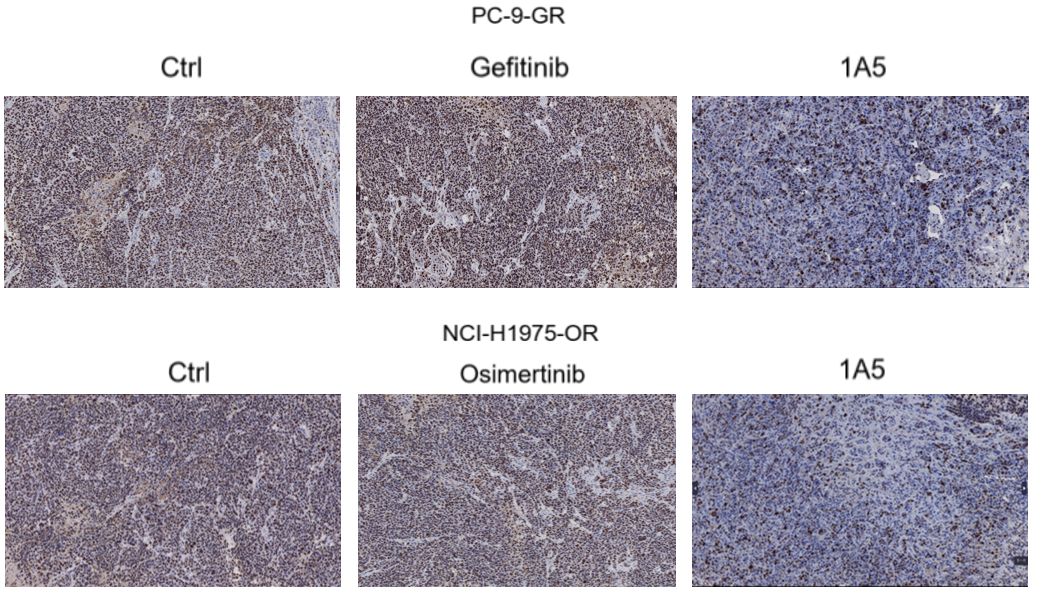


**Figure 6F**


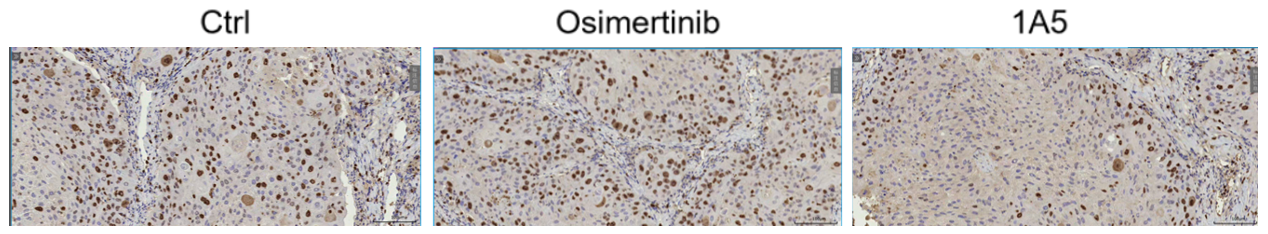

Supplement: Supplementary file 1 — Supporting Information [file ADVS-12-2410945-s001.docx]
